# Supplementary figures and images for: Characterization of a novel interaction of the Nup159 nucleoporin with asymmetrically localized spindle pole body proteins and its link with autophagy
Source: PLoS Biol. 2023 Aug 3;21(8):e3002224. doi: 10.1371/journal.pbio.3002224 (PMC10437821; doi:10.1371/journal.pbio.3002224)

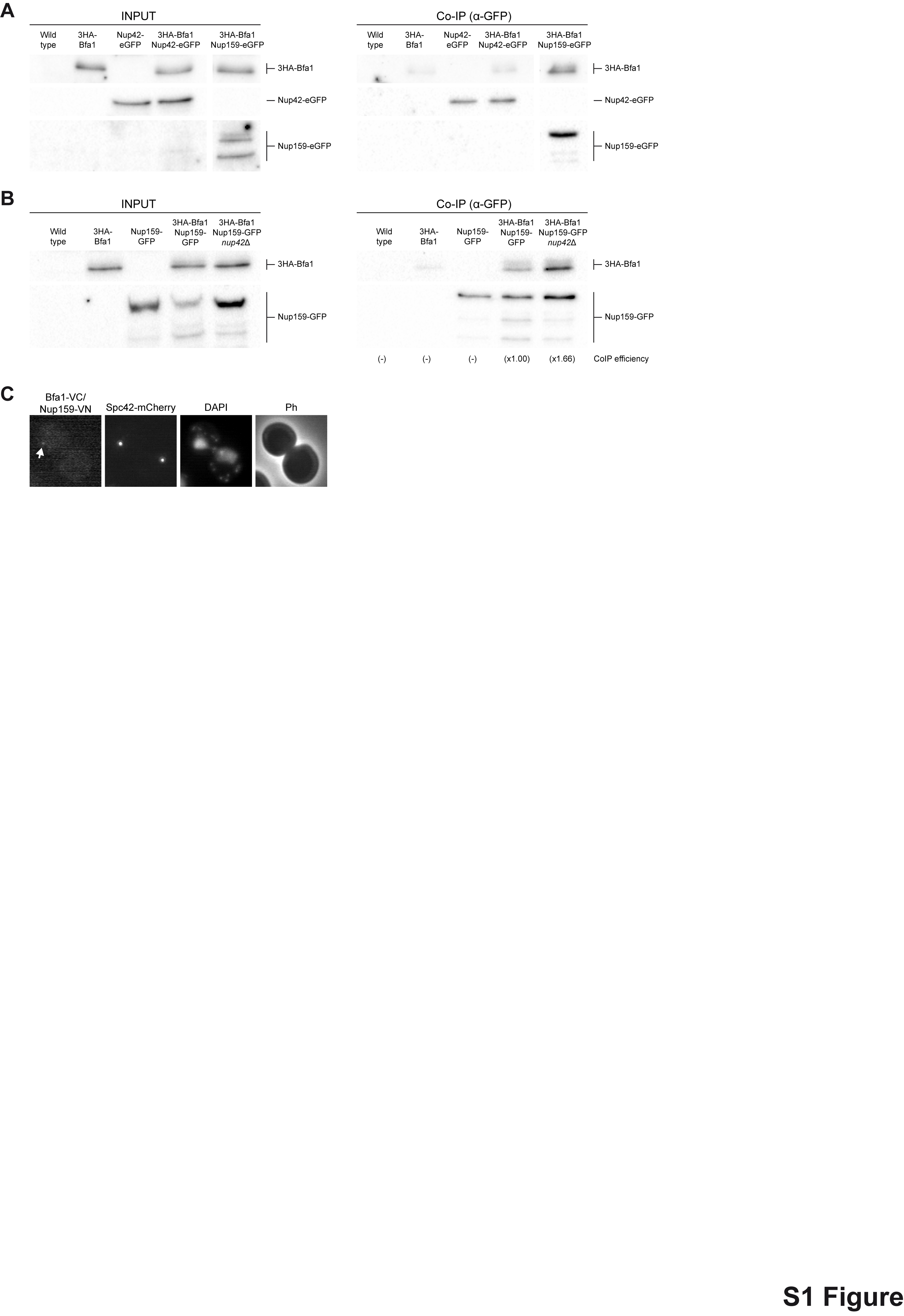

Supplement: S1 Fig — (A, B) Co-immunoprecipitation analysis in cells simultaneously expressing 3HA-Bfa1 and Nup159-eGFP, both in a nup42Δ or in an otherwise wild-type background, as well as in cells from another strain that concurrently expresses 3HA-Bfa1 and Nup42-eGFP. Cells expressing only 3HA-Bfa1, Nup159-eGFP, or Nup42-eGFP, as well as the wild-type strain, were also included as controls. Stationary phase cultures in YPAD were diluted to OD600 = 0.2 in fresh medium and grown for 6 h at 26°C. Western blot gel images for 3HA-Bfa1, Nup159-eGFP, and/or Nup42-eGFP are shown for both the input (INPUT) and the immunoprecipitated (Co-IP) samples. The Co-IP efficiency for 3HA-Bfa1 relative to the corresponding control with untagged Nup159 (-) and referred to the strain used as a reference (×1.00) in (B) is indicated in each case. (C) Gray scale images for each of the individual fluorescent channels in Fig 1C, which displays a positive BiFC interaction between Bfa1-VC and Nup159-VN. (TIF) [file pbio.3002224.s001.tif]

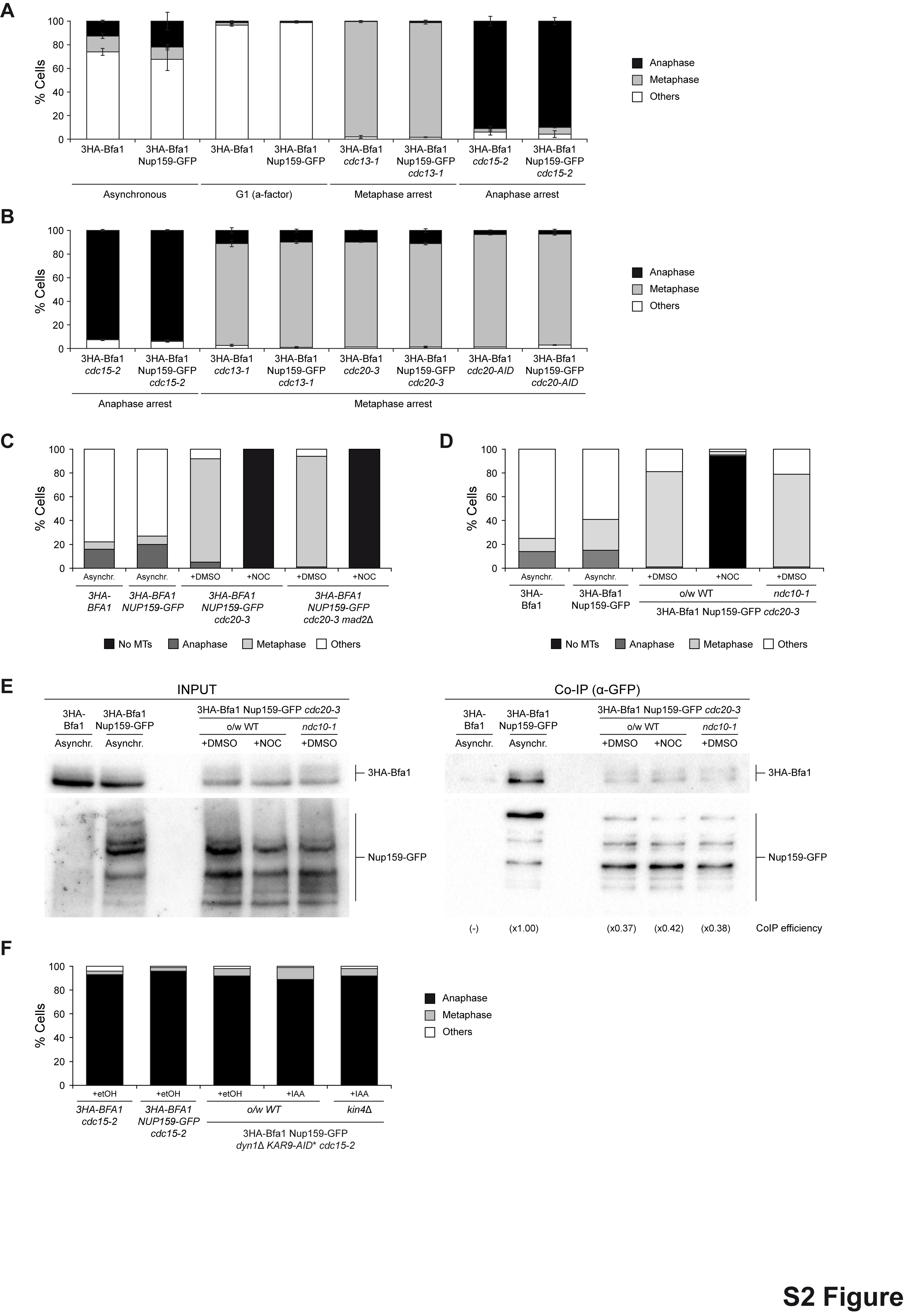

Supplement: S2 Fig — (A, B) Percentage of cells in metaphase, anaphase or other stages of the cell cycle, for the co-immunoprecipitation experiments shown in Fig 2A (A) and Fig 2B (B). Data are available in S1 Data. (C) Percentage of cells that did not display microtubules, as well as those of cells in metaphase, anaphase, or other stages of the cell cycle, for the co-immunoprecipitation experiment shown in Fig 3A. Data are available in S1 Data. (D, E) Co-immunoprecipitation analysis in cells simultaneously expressing 3HA-Bfa1 and Nup159-GFP in a cdc20-3, a cdc20-3 ndc10-1 or an otherwise wild-type background. Cells expressing 3HA-Bfa1 were included as a control. Stationary phase cells in YPAD were diluted to OD600 = 0.2 in fresh medium and either grown in YPAD medium at 26°C for 6 h (Asynchr.) or alternatively arrested in G1 with 5 μg/ml α-factor and then released into YPAD medium at 34°C without pheromone and with (+NOC) or without (+DMSO) 15 μg/ml nocodazole. (D) Percentage of cells that did not display microtubules, as well as those of cells in metaphase, anaphase, or other stages of the cell cycle. Data are available in S1 Data. (E) Western blot gel images for 3HA-Bfa1 and Nup159-GFP for both the input (INPUT) and the immunoprecipitated (Co-IP) samples. The Co-IP efficiency for 3HA-Bfa1 relative to the corresponding control with untagged Nup159 (-) and referred to the strain or condition used as a reference (×1.00) is indicated in each case. (F) Percentage of cells in metaphase, anaphase, or other stages of the cell cycle, for the co-immunoprecipitation experiment shown in Fig 3B–3D. Data are available in S1 Data. (TIF) [file pbio.3002224.s002.tif]

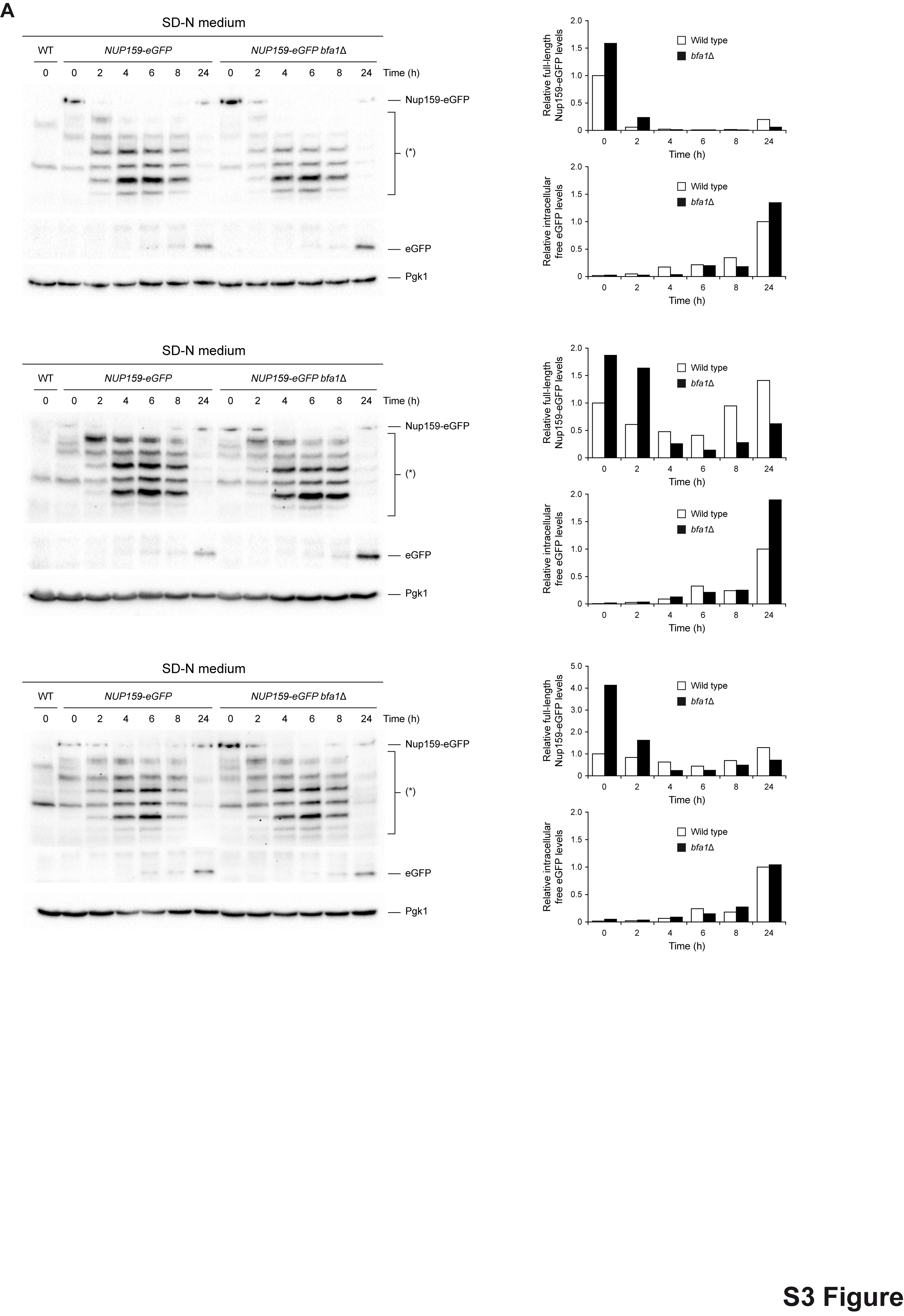

Supplement: S3 Fig — (A) Western blot gel images of the three biological replicates used for the quantifications in Fig 5B and 5C, displaying Nup159-eGFP and free eGFP levels at the indicated time points after cells were transferred to SD-N medium (time = 0 h). Intermediate Nup159-eGFP degradation forms are indicated with (*). Pgk1 was used as a loading control. Graphs showing the quantification of the levels of full-length Nup159-eGFP and free eGFP for each of the experiments are also included next to each western blot image. Data are available in S1 Data. (TIF) [file pbio.3002224.s003.tif]

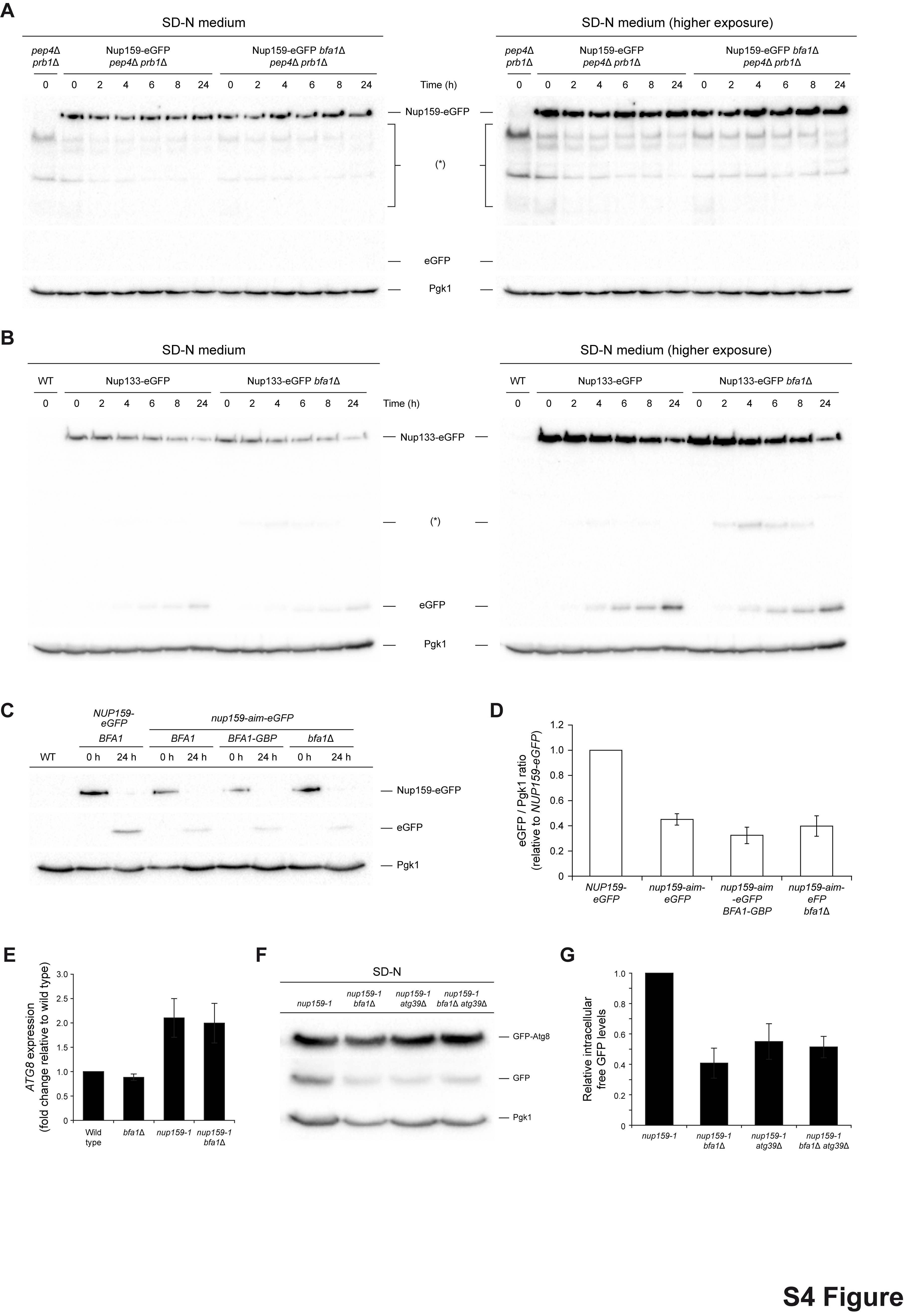

Supplement: S4 Fig — (A–D) Stationary phase cultures in YPAD were diluted to OD600 = 0.2 in SD-N medium and grown for 24 h at 26°C. (A) Western blot gel images displaying Nup159-eGFP and free eGFP levels at the indicated time points are shown for otherwise wild-type, NUP159-eGFP and NUP159-eGFP bfa1Δ cells, all further carrying PEP4 and PRB1 gene deletions, after being transferred to SD-N medium (time = 0 h). Intermediate Nup159-eGFP degradation forms are indicated with (*). Pgk1 was used as a loading control. To facilitate visualization of the fainter bands overexposed images of the same gels are also shown (higher exposure). Experiment was carried out thrice (n = 3) and a representative image is shown. (B) Western blot gel images displaying Nup133-eGFP and free eGFP levels at the indicated time points are shown for wild type, NUP133-eGFP and NUP133-eGFP bfa1Δ cells after being transferred to SD-N medium (time = 0 h). Intermediate Nup133-eGFP degradation forms are indicated with (*). Pgk1 was used as a loading control. To facilitate visualization of the fainter bands overexposed images of the same gels are also shown (higher exposure). Experiment was carried out thrice (n = 3) and a representative image is shown. (C) Western blot gel images displaying Nup159-eGFP in wild-type cells, as well as levels of Nup159-AIM-eGFP in cells expressing Bfa1-GBP, in a bfa1Δ mutant or in an otherwise wild-type background, 24 h after being transferred to SD-N medium. Pgk1 was used as a loading control. Experiment was carried out thrice (n = 3) and a representative image is shown. (D) Quantification of the relative levels of free eGFP in (C). Data are the average of 5 experiments (n = 5) and are available in S1 Data. Error bars represent SEM. (E) ATG8 gene expression determined by quantitative RT-PCR in the indicated strains and normalized to the wild type. Data are the average of 3 experiments (n = 3) and are available in S1 Data. Error bars represent SEM. (F) Western blot gel images displaying GFP-Atg [file pbio.3002224.s004.tif]

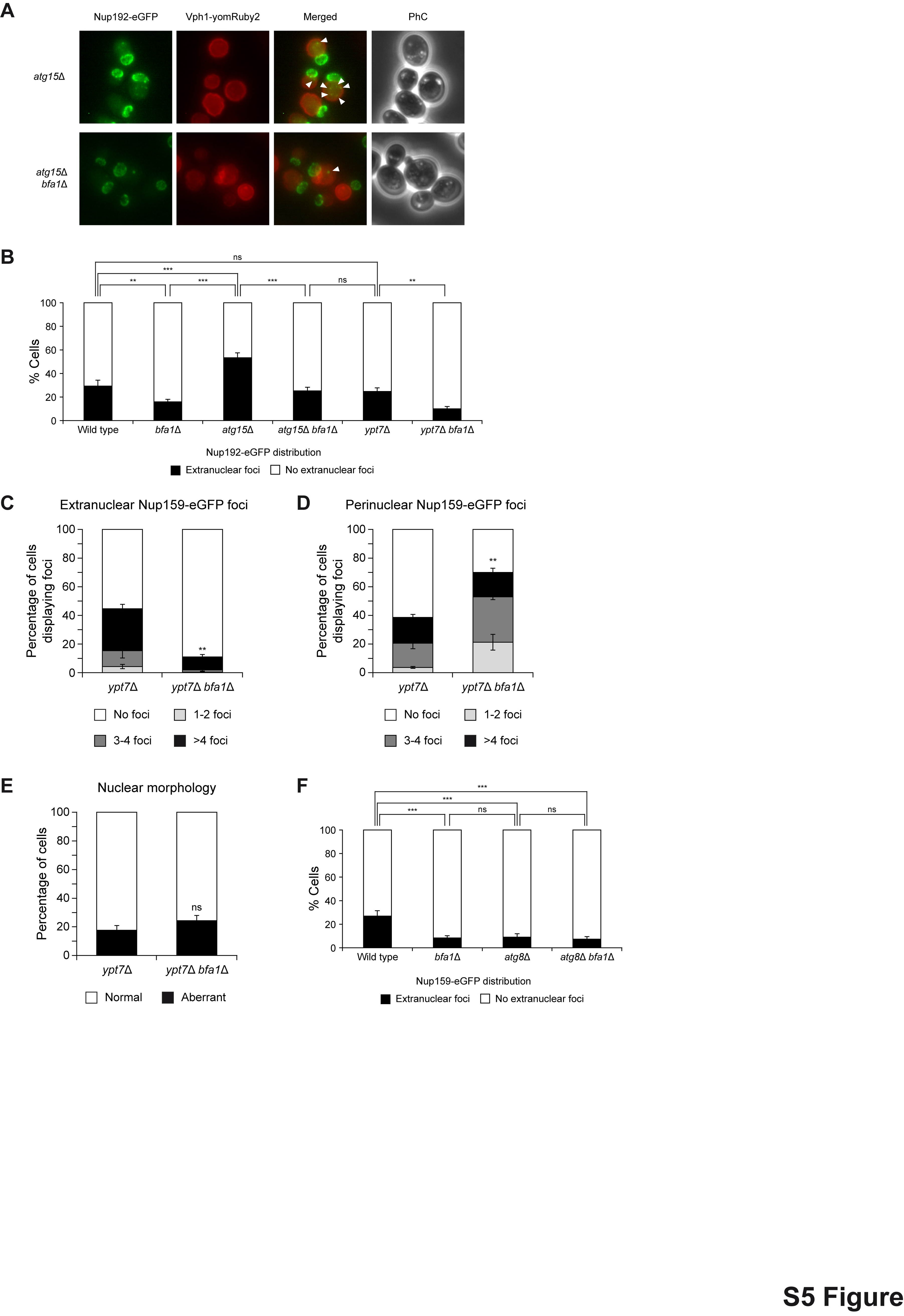

Supplement: S5 Fig — (A–F) Stationary phase cultures in YPAD were diluted to OD600 = 0.2 in SD-N medium and grown for 4 h (C–F) or 24 h (A, B) at 26°C. (A) Representative images of live cells expressing Nup192-eGFP (green) and Vph1-yomRuby2 (red) in atg15Δ and atg15Δ bfa1Δ cells. Phase-contrast (PhC) and merged images are also shown. (B) Quantification of the percentage of cells displaying (black bars) or not (white bars) extranuclear Nup192-eGFP foci. Data are the average of 3 experiments (n = 3; 100 cells/each) and are available in S1 Data. Error bars represent SD. (C–E) Quantification of the percentage of cells displaying extranuclear (C) and perinuclear (D) Nup159-eGFP foci, as well as of cells displaying an aberrant nuclear morphology (E), 4 h after being transferred to SD-N medium. An estimation of the percentage of cells displaying 1–2, 3–4, or more than 4 foci is also shown for both cells with extranuclear (C) and perinuclear (D) Nup159-eGFP clusters. Data are the average of 3 experiments (n = 3; 100 cells/each) and are available in S1 Data. Error bars represent SEM. (F) Quantification of the percentage of cells displaying (black bars) or not (white bars) extranuclear Nup159-eGFP foci. Data are the average of 3 experiments (n = 3; 100 cells/each) and are available in S1 Data. Error bars represent SD. (TIF) [file pbio.3002224.s005.tif]
