# Supplementary material for: Characterization of a novel interaction of the Nup159 nucleoporin with asymmetrically localized spindle pole body proteins and its link with autophagy
Source: PLoS Biol. 2023 Aug 3;21(8):e3002224. doi: 10.1371/journal.pbio.3002224 (PMC10437821; doi:10.1371/journal.pbio.3002224)
Supplement: S1 Table — List of the strains in this study, which also details the specific experiment in which each of the strains was used. (DOCX) [file pbio.3002224.s006.docx]

**S1 Table: Strains**

| Strain | Relevant genotype | Experiment |
| --- | --- | --- |
| F496 | MATa (wild type from the W303 background) | Figs 1A, 5A, 5D, 5F, 6A-6C, S1A-S1B, S3A, S4B-S4C and S4E |
| F25 | MATa, *bub2::BUB2-3HA* | Fig 1B |
| F505 | MATa, *bfa1::3HA-BFA1* | Figs 1A-1B, 2A, 3A, 4A-4B, S1A-S1B, S2A and S2C-S2E |
| F1114 | MATa, *bfa1::KanMX6* | S4E Fig |
| F1492 | MATa, *bfa1::3HA-BFA1, cdc15-2* | Figs 2A-2B, 3C-3F, S2A-S2B and S2F |
| F2640 | MATa, *spc42::SPC42-mcherry-KanMX, bfa1::BFA1-VC-KanMX, nup42::NUP42-VN-His3MX6* | Fig 1D |
| F2847 | MATa, *nup159::NUP159-GFP-TRP1* | Figs 1A-1B, 5E, and S1B |
| F2848 | MATa, *bfa1::3HA-BFA1, nup159::NUP159-GFP-TRP1* | Figs 1A-1B, 2A, 3A, 4A-4B, S1B, S2A and S2C-S2E |
| F2881 | MATa, *bfa1::3HA-BFA1, nup159::NUP159-GFP-TRP1, bub2::HIS3MX6* | Fig 1A |
| F2882 | MATa, *bfa1::3HA-BFA1, nup159::NUP159-GFP-TRP1, cdc15-2* | Figs 2A-2B, 3C-3F, S2A-S2B and S2F |
| F2892 | MATa, *bfa1::3HA-BFA1, nup159::NUP159-GFP-TRP1, cdc13-1* | Figs 2A-2B, S2A-S2B |
| F2894 | MATa, *bfa1::3HA-BFA1, cdc13-1* | Figs 2A-2B and S2A-S2B |
| F2956 | MATa, *bfa1::3HA-BFA1, nup159::NUP159-GFP-TRP1, cdc20::CDC20-AID-KanMX* | Figs 2B and S2B |
| F2958 | MATa, *bfa1::3HA-BFA1, cdc20::CDC20-AID-KanMX* | Figs 2B and S2B |
| F3109 | MATa, *bub2::BUB2-3HA, nup159::NUP159-GFP-TRP1* | Fig 1B |
| F3237 | MATa, *nup42::NUP42-yEGFP-SpHIS5* | S1A Fig |
| F3238 | MATa, *bfa1::3HA-BFA1, nup42::NUP42-yEGFP-SpHIS5* | S1A Fig |
| F3294 | MATa, *bfa1::3HA-BFA1, nup159::NUP159-yEGFP-HisMX6* | S1A Fig |
| F3610 | MATa, *bfa1::3HA-BFA1, cdc20-3* | Figs 2B and S2B |
| F3612 | MATa, *bfa1::3HA-BFA1, nup159::NUP159-GFP-TRP1, cdc20-3* | Figs 2B, 3A and S2B-S2E |
| F3802 | MATa, *bfa1::3HA-BFA1, nup159::NUP159-GFP-TRP1, cdc20-3, mad2::KanMX6* | Figs 3A and S2C |
| F3814 | MATa, *bfa1::3HA-BFA1, nup159::NUP159-GFP-TRP1, cdc20-3, ndc10-1* | S2D-S2E Fig |
| F3826 | MATa, *bfa1::3HA-BFA1, nup159::NUP159-GFP-TRP1, cdc15-2, dyn1::URA3, kar9::KAR9-AID*-9myc-KanMX6, ura3-1::ADH1-OsTIR1-9Myc-URA3* | Figs 3B-3D and S2F |
| F3844 | MATa, *bfa1::3HA-BFA1, nup159::NUP159-GFP-TRP1, nup42::TRP1* | S1B Fig |
| F3982 | MATa, *bfa1::3HA-BFA1, nup159::NUP159-GFP-TRP1, cdc15-2, dyn1::URA3, kar9::KAR9-AID*-9myc-KanMX6, ura3-1::ADH1-OsTIR1-9Myc-URA3, kin4::KanMX6* | Figs 3C-3D and S2F |
| F4254 | MATa, *nup159::nup159-1-GFP-HisMX6* | Fig 5E-5F |
| F4255 | MATa, *nup159::nup159-1-3HA-HisMX6* | S4E Fig |
| F4340 | MATa, *bfa1::3HA-BFA1, nup159::nup159-1-GFP-HixMX6* | Fig 4A |
| F4344 | MATa, *bfa1::3HA-BFA1, dyn2::HisMX6* | Fig 4B |
| F4346 | MATa, *nup159::NUP159-GFP-TRP1, dyn2::HisMX6* | Fig 4B |
| F4348 | MATa, *bfa1::3HA-BFA1, nup159::NUP159-GFP-TRP1, dyn2::HisMX6* | Fig 4B |
| F4463 | MATa, *bfa1::3HA-BFA1, nup159::NUP159-GFP-TRP1, cdc5-2-URA3* | Fig 3E-3F |
| F4558 | MATa, *ura3::pRS306-BFA1::URA3* | Figs 4D-4E and 4I-4J |
| F4561 | MATa, *ura3::pRS306-BFA1::URA3, bfa1::BFA1-yeGFP-kanMX6, nup159::NUP159-GBP-KanMX6* | Figs 4C, 4F-4G and 4I-4J |
| F4596 | MATa, *nup159::nup159-1-GFP-HisMX6, bfa1::KanMX6* | Fig 5E-5F |
| F5160 | MATa, *nup159::NUP159-GFP-TRP1, bfa1::KanMX6* | Fig 5E |
| F5205 | MATa, *nup133::NUP133-yEGFP-HisMX6* | S4B Fig |
| F5206 | MATa, *nup133::NUP133-yEGFP*, *bfa1::KanMX6* | S4B Fig |
| F5229 | MATa, *nup159::NUP159-yEGFP-HisMX6* | Figs 5A-5C, S3A and S4C-S4D |
| F5266 | MATa, *prb1::hphMX4, pep4::NatMx6* | S4A Fig |
| F5286 | MATa, *nup159::NUP159-yEGFP-HisMX6*, *bfa1::KanMX6* | Figs 5A-5C and S3A |
| F5329 | MATa, *nup159::NUP159-yEGFP-HisMX6, prb1::hphMX4, pep4::NatMx6* | S4A Fig |
| F5331 | MATa, *nup159::NUP159-yEGFP-HisMX6*, *bfa1::KanMX6, prb1::hphMX4, pep4::NatMx6* | S4A Fig |
| F5343 | MATa, *nup159::NUP159-3HA-HisMX6*, *pCUP1-GFP-ATG8-URA3* | Fig 5D |
| F5345 | MATa, *nup159::NUP159-3HA-HisMX6*, *pCUP1-GFP-ATG8-URA3*, *bfa1::KanMX6* | Fig 5D |
| F5375 | MATa, *nup159::NUP159-yEGFP-HisMX6, vph1::VPH1-yomRuby2-His3Mx6* | Figs 6F-6I and S5F |
| F5376 | MATa, *nup159::NUP159-yEGFP-HisMX6, vph1::VPH1-yomRuby2-His3Mx6*, *bfa1::KanMX6* | Figs 6F-6I and S5F |
| F5381 | MATa, *spc42::SPC42-mcherry-KanMX, bfa1::BFA1-VC-KanMX, nup159::NUP159-VN-His3MX6* | Figs 1C-1D, 2C-2D and S1C |
| F5455 | MATa, *nup159::nup159-1-GFP-HisMX6, atg39::natMX6* | Fig 5F |
| F5457 | MATa, *nup159::nup159-1-GFP-HisMX6, bfa1::KanMX6, atg39::natMX6* | Fig 5F |
| F5523 | MATa, *spc42::SPC42-mcherry-KanMX, bfa1::BFA1-VC-KanMX, nup159::NUP159-VN-His3MX6, cdc15-2* | Fig 2C-2D |
| F5556 | MATa, *spc42::SPC42-mcherry-KanMX, bfa1::BFA1-VC-KanMX, nup159::NUP159-VN-His3MX6, cdc13-1* | Fig 2C-2D |
| F5558 | MATa, *spc42::SPC42-mcherry-KanMX, bfa1::BFA1-VC-KanMX, nup159::NUP159-VN-His3MX6, cdc20-3* | Fig 2C-2D |
| F5575 | MATa, *nup159::nup159-AIM-yEGFP-HisMX6* | S4C-S4D Fig |
| F5580 | MATa, *nup192::NUP192-yEGFP-HisMX6, vph1::VPH1-yomRuby2-His3Mx6* | S5B Fig |
| F5581 | MATa, *nup192::NUP192-yEGFP-HisMX6, vph1::VPH1-yomRuby2-His3Mx6*, *bfa1::KanMX6* | S5B Fig |
| F5582 | MATa, *nup192::NUP192-yEGFP-HisMX6, vph1::VPH1-yomRuby2-His3Mx6*, *atg15::NatMX* | S5A-S5B Fig |
| F5583 | MATa, *nup192::NUP192-yEGFP-HisMX6, vph1::VPH1-yomRuby2-His3Mx6*, *atg15::NatMX, bfa1::KanMX6* | S5A-S5B Fig |
| F5586 | MATa, *pCUP1-GFP-ATG8-URA3, vph1::VPH1-yomRuby2-His3Mx6*, *atg15::NatMX* | Fig 6E |
| F5587 | MATa, *pCUP1-GFP-ATG8-URA3, vph1::VPH1-yomRuby2-His3Mx6*, *atg15::NatMX, bfa1::KanMX6* | Fig 6E |
| F5590 | MATa, *nup159::nup159-1-3HA-HisMX6, bfa1::KanMX6* | S4E Fig |
| F5596 | MATa, *nup159::nup159-1-3HA-HisMX6, pCUP1-GFP-ATG8-URA3* | Figs 6A-6C and S4F-S4G |
| F5598 | MATa, *nup159::nup159-1-3HA-HisMX6, pCUP1-GFP-ATG8-URA3, bfa1::KanMX6* | Fig 6A-C and S4F-S4G |
| F5600 | MATa, *nup159::nup159-1-3HA-HisMX6, pCUP1-GFP-ATG8-URA3, prb1::hphMX4, pep4::NatMx6* | Fig 6A-6C |
| F5602 | MATa, *nup159::nup159-1-3HA-HisMX6, pCUP1-GFP-ATG8-URA3, bfa1::KanMX6, prb1::hphMX4, pep4::NatMx6* | Fig 6A-6C |
| F5614 | MATa, *nup159::nup159-AIM-yEGFP-HisMX6, bfa1::BFA1-GBP-KanMX4* | S4C-S4D Fig |
| F5616 | MATa, *nup159::nup159-AIM-yEGFP-HisMX6, bfa1::KanMX6* | S4C-S4D Fig |
| F5669 | MATa, *nup192::NUP192-yEGFP-HisMX6, vph1::VPH1-yomRuby2-His3Mx6*, *ypt7::NatMX* | S5B Fig |
| F5670 | MATa, *nup192::NUP192-yEGFP-HisMX6, vph1::VPH1-yomRuby2-His3Mx6*, *ypt7::NatMX, bfa1::KanMX6* | S5B Fig |
| F5677 | MATa, *nup159::nup159-1-3HA-HisMX6*, *pCUP1-GFP-ATG8-URA3, vph1::VPH1-yomRuby2-His3Mx6*, *atg15::NatMX* | Fig 6D-6E |
| F5678 | MATa, *nup159::nup159-1-3HA-HisMX6*, *pCUP1-GFP-ATG8-URA3, vph1::VPH1-yomRuby2-His3Mx6*, *atg15::NatMX, bfa1::KanMX6* | Fig 6D-6E |
| F5680 | MATa, *nup159::NUP159-yEGFP-HisMX6, vph1::VPH1-yomRuby2-His3Mx6*, *ypt7::NatMX* | S5C-S5E Fig |
| F5682 | MATa, *nup159::NUP159-yEGFP-HisMX6, vph1::VPH1-yomRuby2-His3Mx6*, *ypt7::NatMX, bfa1::KanMX6* | S5C-S5E Fig |
| F5688 | MATa, *spc110::SPC110-dsRed-KanMX6, bfa1::BFA1-VC-KanMX, nup159::NUP159-VN-His3MX6* | Fig 1D |
| F5704 | MATa, *spc110::SPC110-dsRed-KanMX6, bfa1::BFA1-VC-KanMX, nup100::NUP100-VN-His3MX6* | Fig 1D |
| F5706 | MATa, *nup159::NUP159-yEGFP-HisMX6, vph1::VPH1-yomRuby2-His3Mx6, atg8::NatMX6* | S5F Fig |
| F5708 | MATa, *nup159::NUP159-yEGFP-HisMX6, vph1::VPH1-yomRuby2-His3Mx6, atg8::NatMX6, bfa1::KanMX6* | S5F Fig |
| F5710 | MATa, *spc110::SPC110-dsRed-KanMX6, bfa1::BFA1-VC-KanMX, dyn2::DYN2-VN-His3MX6* | Fig 1D |
| F5714 | MATa, *spc110::SPC110-dsRed-KanMX6, bfa1::BFA1-VC-KanMX, gle1::GLE1-VN-His3MX6* | Fig 1D |
| F5857 | MATa, *nup159::nup159-1-3HA-HisMX6, pCUP1-GFP-ATG8-URA3, atg39::NatMX6* | S4F-S4G Fig |
| F5858 | MATa, *nup159::nup159-1-3HA-HisMX6, pCUP1-GFP-ATG8-URA3, atg39::NatMX6, bfa1::KanMX6* | S4F-S4G Fig |
