# Supplementary material for: Characterization of a novel interaction of the Nup159 nucleoporin with asymmetrically localized spindle pole body proteins and its link with autophagy
Source: PLoS Biol. 2023 Aug 3;21(8):e3002224. doi: 10.1371/journal.pbio.3002224 (PMC10437821; doi:10.1371/journal.pbio.3002224)
Supplement: S2 Table — List of the antibodies used in this study, both for immunofluorescence and western blot analyses. (DOCX) [file pbio.3002224.s007.docx]

**S2 Table: Antibodies for immunofluorescence and Western blot**

| Protein/ epitope | Primary antibody | Primary ab dilution | Secondary antibody | Secondary ab dilution | Technique |
| --- | --- | --- | --- | --- | --- |
| HA | HA.11  (Covance) | 1:2000 | Anti-mouse  HRP-linked  (GE Healthcare) | 1:5000 | Western  blot |
| GFP | JL8 Living Colors (Clontech) | 1:1000 | Anti-mouse  HRP-linked  (GE Healthcare) | 1:3000 | Western  Blot |
| Clb2 | Anti-Clb2  (Santa Cruz) | 1:2000 | Anti-rabbit  HRP-linked  (GE Healthcare) | 1:10000 | Western  Blot |
| Pgk1 | Anti-Pgk1  (Invitrogen) | 1:10000 | Anti-mouse  HRP-linked  (GE Healthcare) | 1:10000 | Western  Blot |
| Tubulin | Anti-tubulin  (Abcam) | 1:250 | Anti-rat FITC  (Jackson  ImmunoResearch) | 1:250 | Immuno-fluorescence |
