# Supplementary material for: Characterization of a novel interaction of the Nup159 nucleoporin with asymmetrically localized spindle pole body proteins and its link with autophagy
Source: PLoS Biol. 2023 Aug 3;21(8):e3002224. doi: 10.1371/journal.pbio.3002224 (PMC10437821; doi:10.1371/journal.pbio.3002224)
Supplement: S3 Table — List of primers used in this study for the analysis of gene expression by quantitative RT-PCR. (DOCX) [file pbio.3002224.s008.docx]

**S3 Table: Oligonucleotide sequences for quantitative RT-PCR**

| Locus | Primer name | Sequence (5’ → 3’) |
| --- | --- | --- |
| *ATG8* | *ATG*8-qPCR-fwd (prF1547) | CGTAAATATCTAGTTCCTGCTGA |
|  | *ATG*8-qPCR-rvs (prF1548) | TTGTGTTCTTGATATATGGCAGA |
| *ACT1* | *ACT1*-RT-PCR-fwd1 (prF897) | TACGCTGGTTTCTCTCTACCTCACGCCATT |
|  | *ACT1*-RT-PCR-rvs1 (prF898) | TTGATGTCACGGACAATTTCTCTTTCAGCA |
