# Supplementary material for: Characterization of a novel interaction of the Nup159 nucleoporin with asymmetrically localized spindle pole body proteins and its link with autophagy
Source: PLoS Biol. 2023 Aug 3;21(8):e3002224. doi: 10.1371/journal.pbio.3002224 (PMC10437821; doi:10.1371/journal.pbio.3002224)
Supplement: S1 Raw Images — Original images of all blots displayed in this study. (PDF) [file pbio.3002224.s009.pdf]

Figure 1A: Original blot images

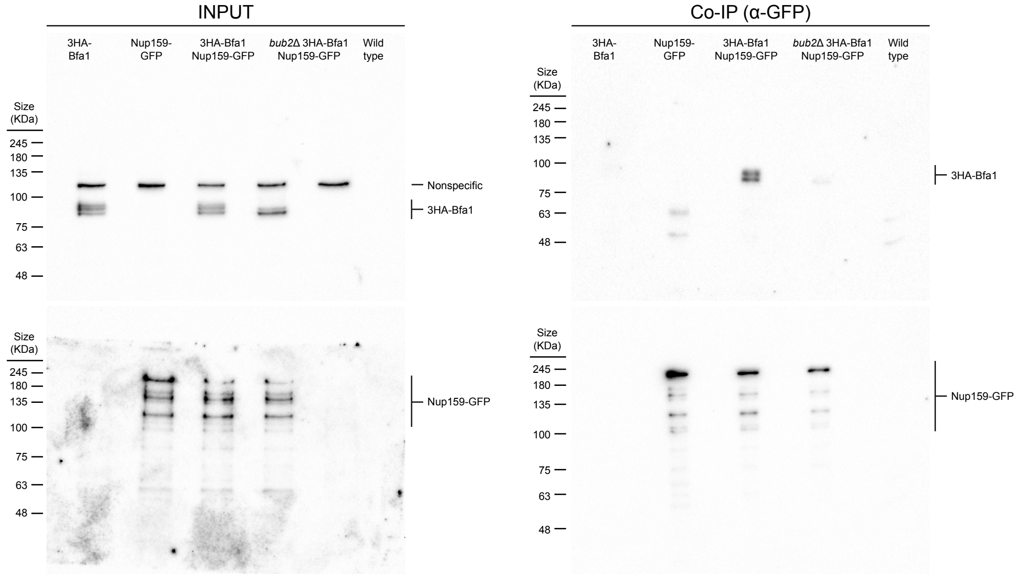

Figure 1B: Original blot images

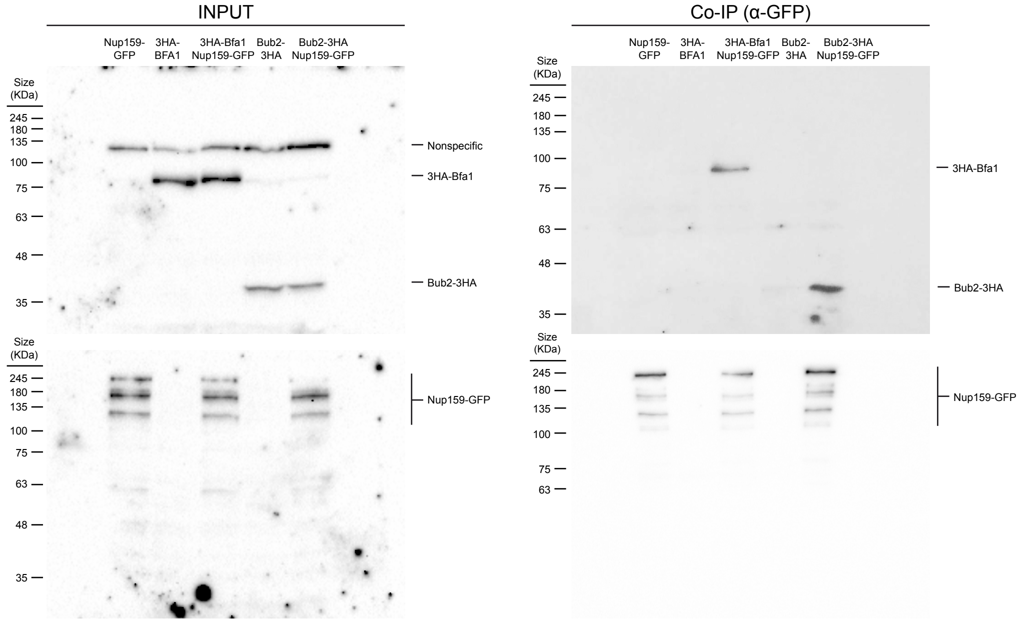

Figure 2A: Original blot images

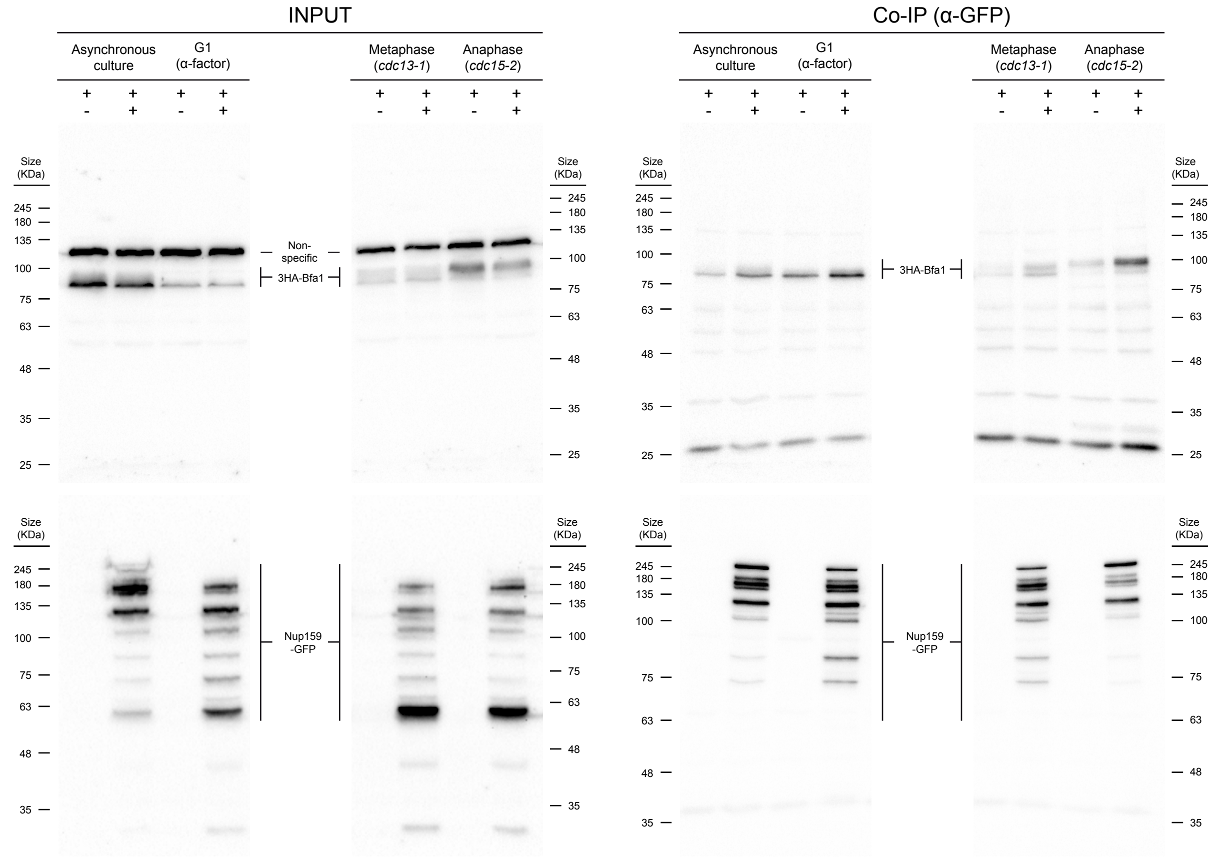

Figure 2B: Original blot images

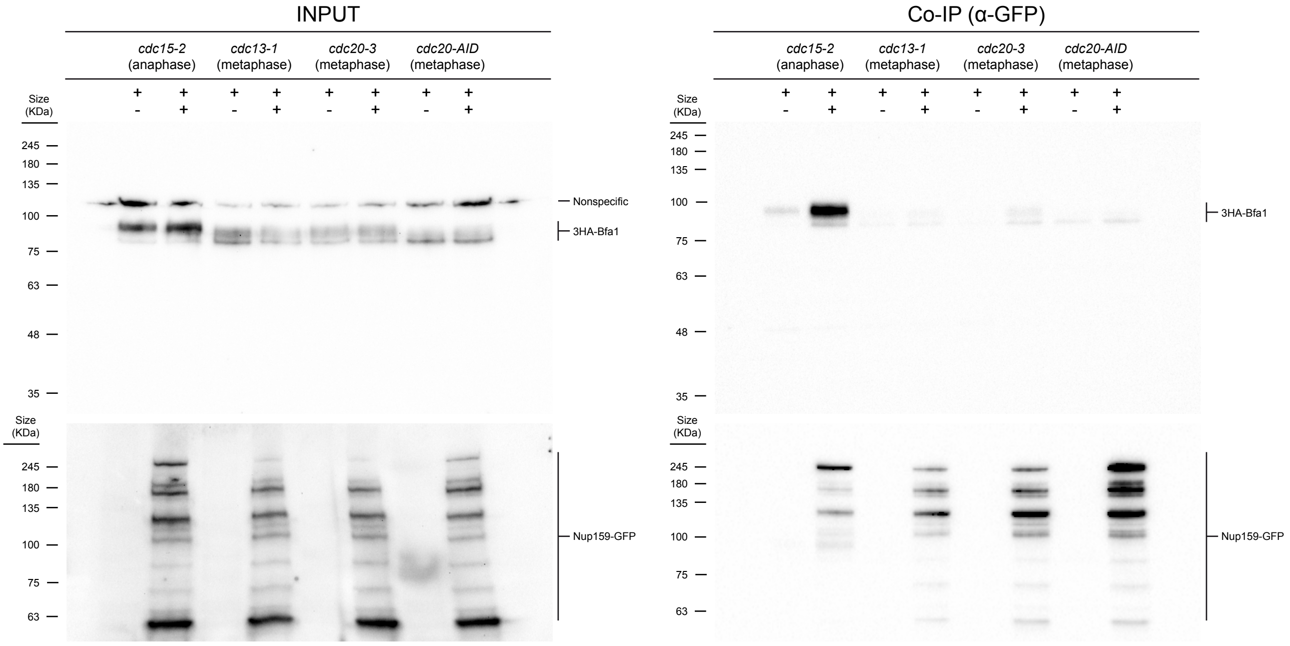

Raw images 2

Figure 3A: Original blot images

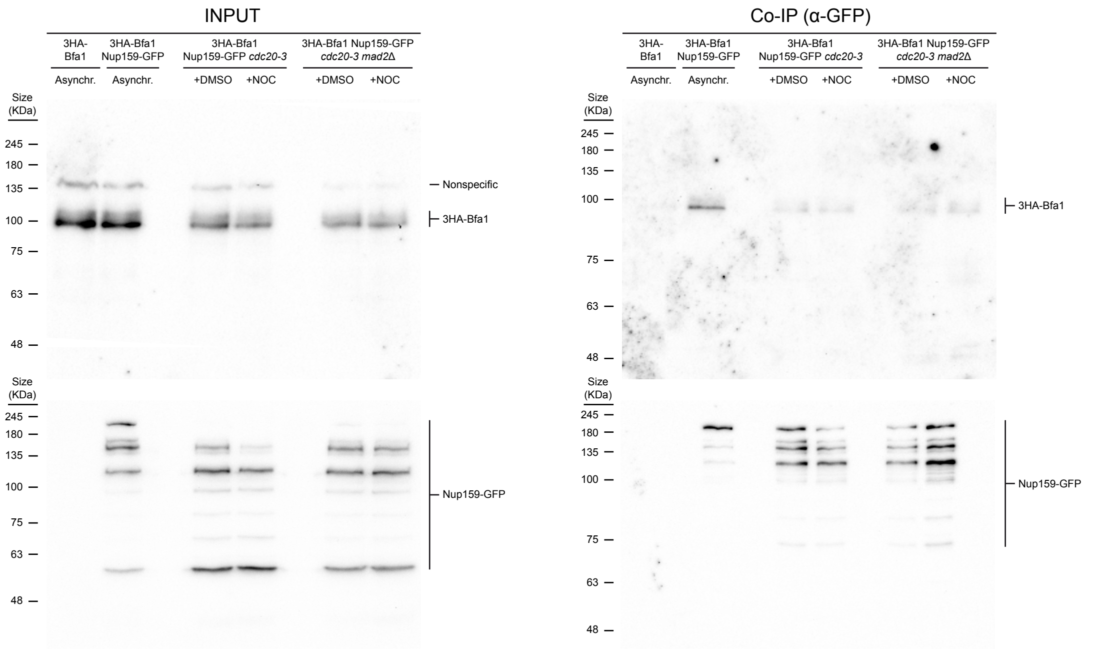

Figure 3D: Original blot images

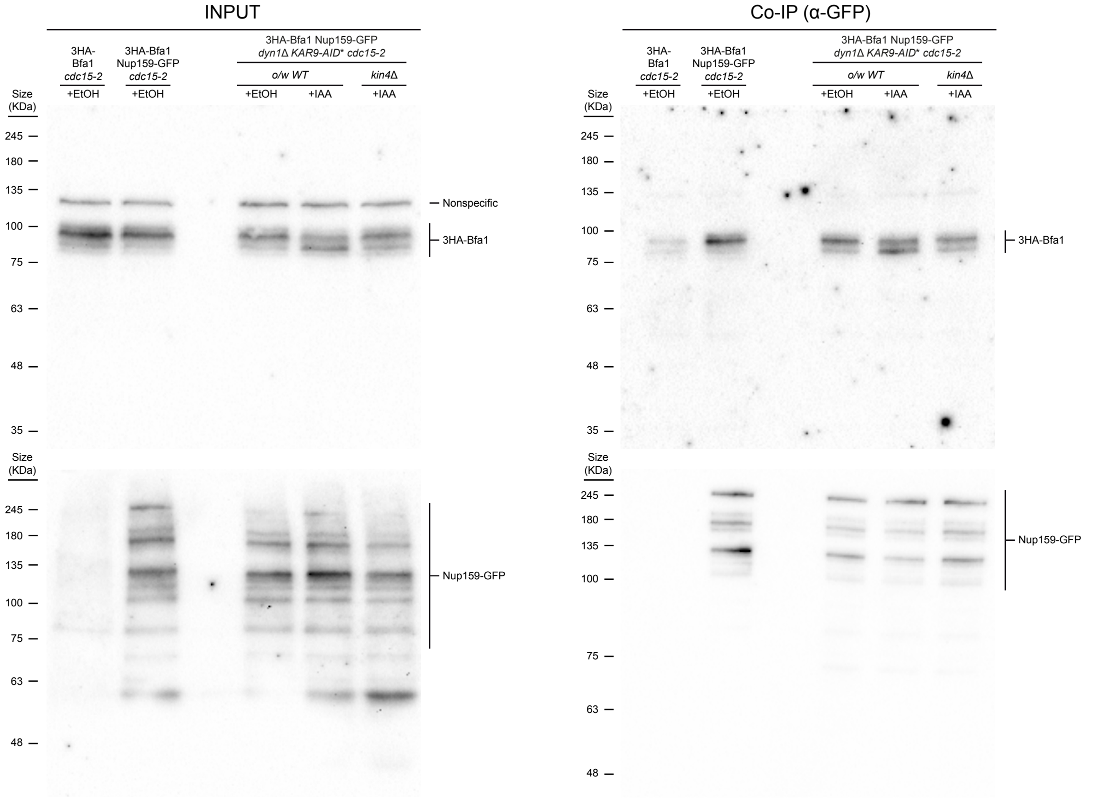

Figure 3F: Original blot images

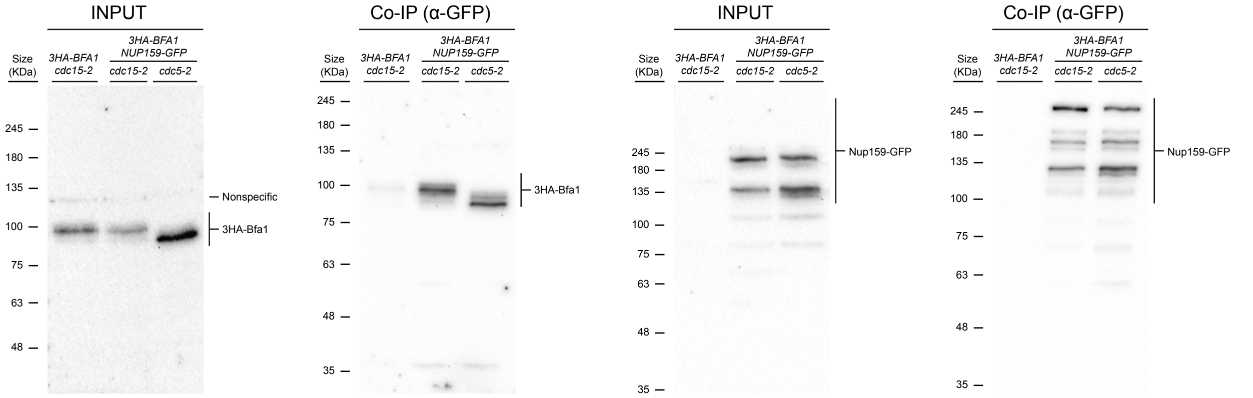

Raw images 3

Figure 4A: Original blot images

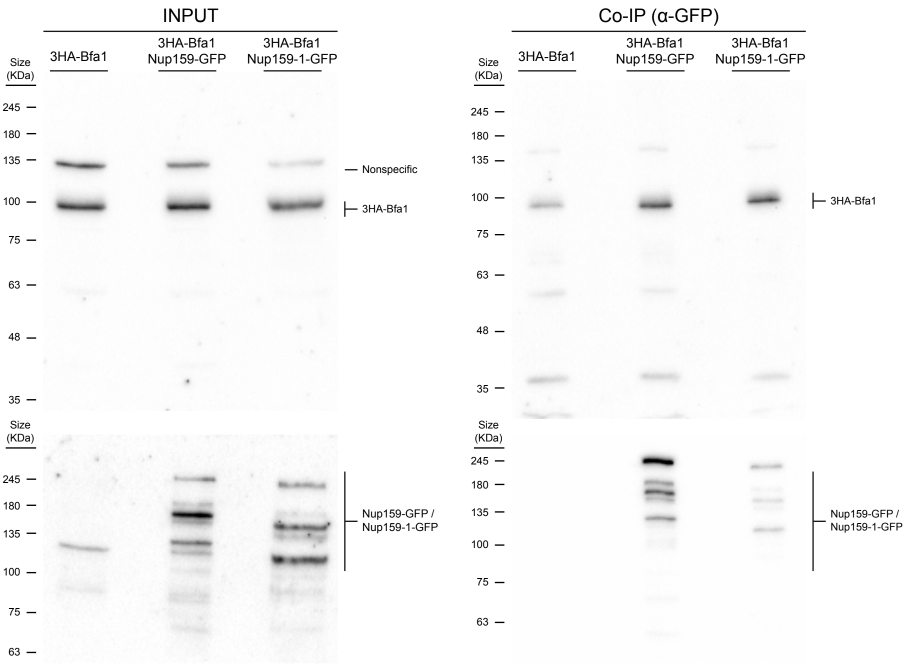

Figure 4B: Original blot images

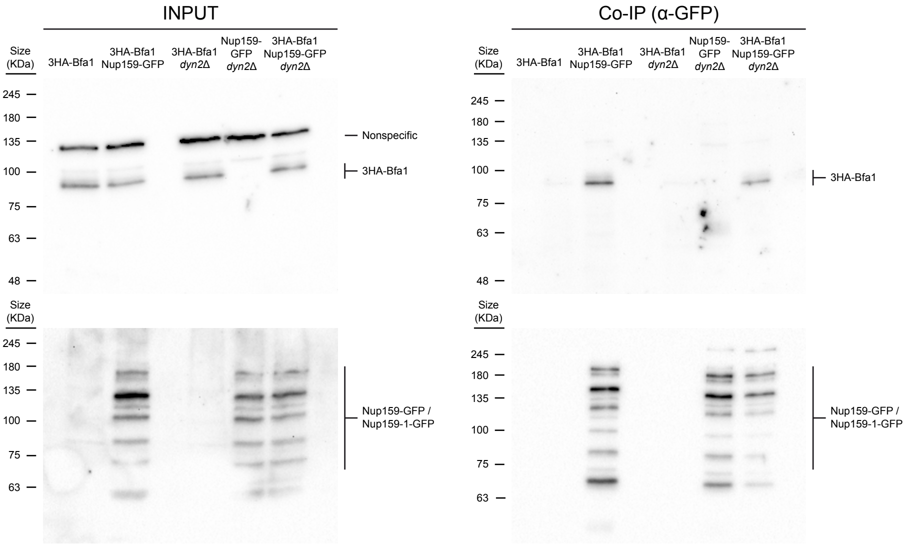

Figure 4E: Original blot images

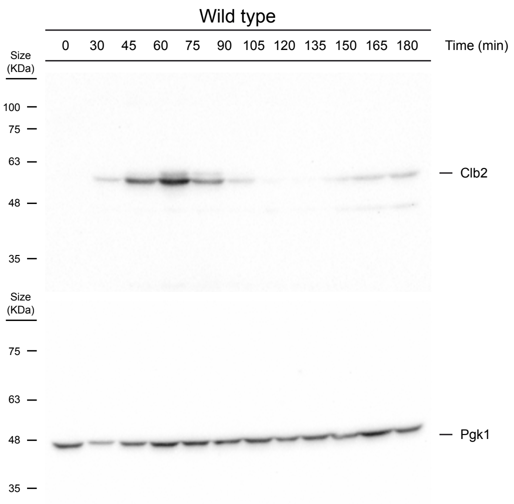

Figure 4G: Original blot images

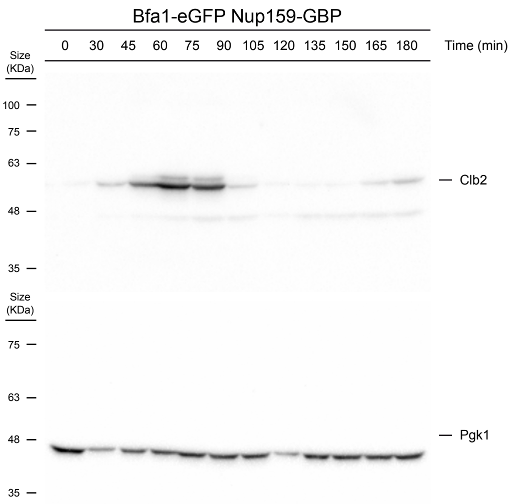

Figure 5A and Figure S3: Original blot images

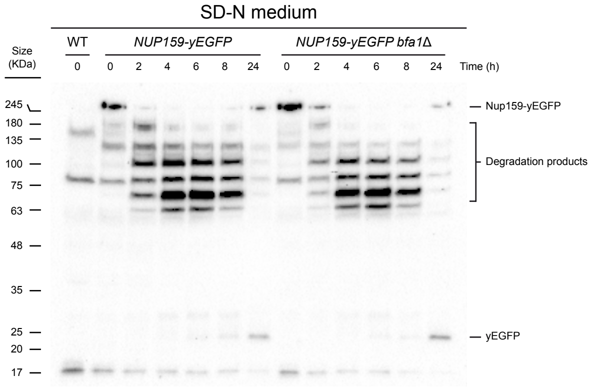

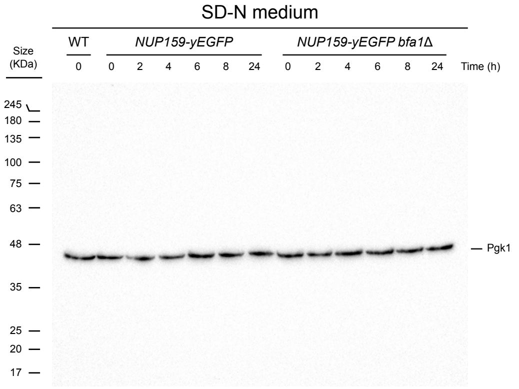

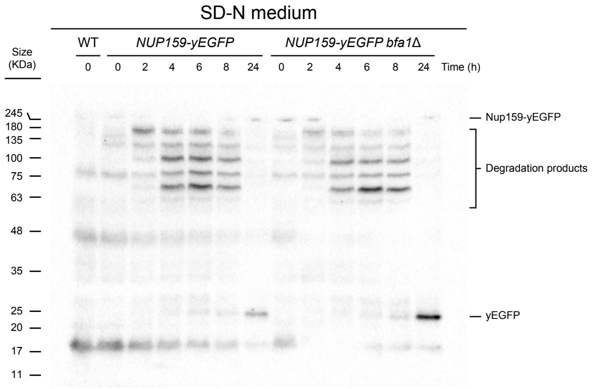

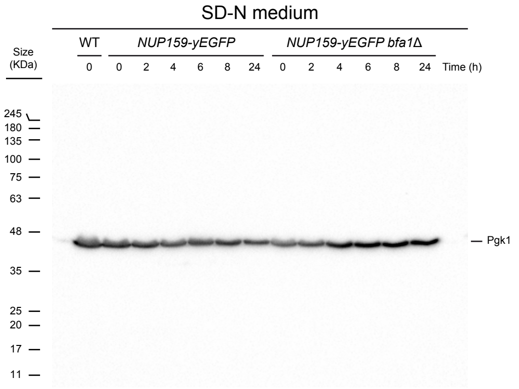

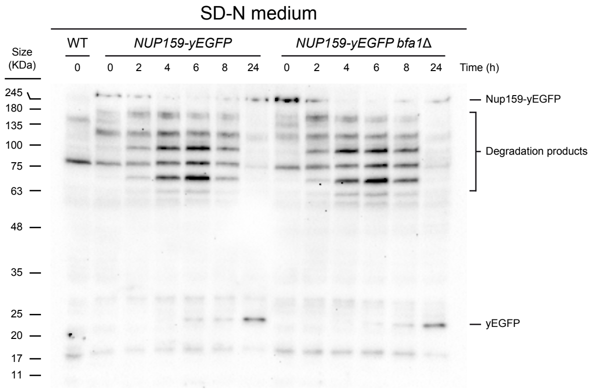

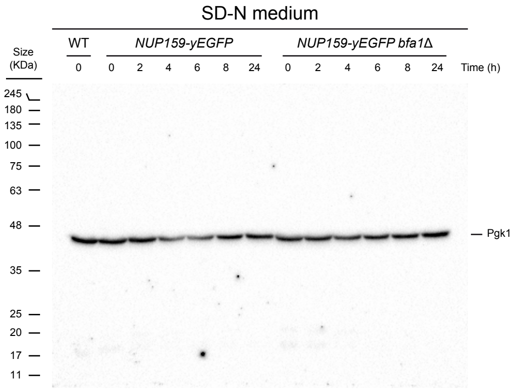

Figure 5D: Original blot images

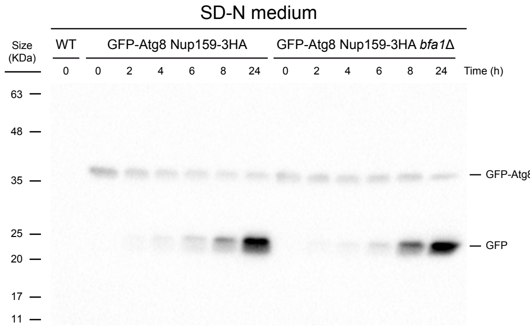

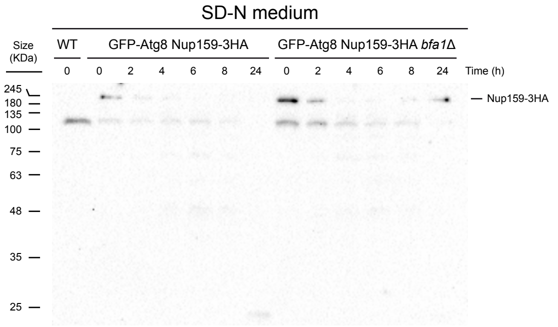

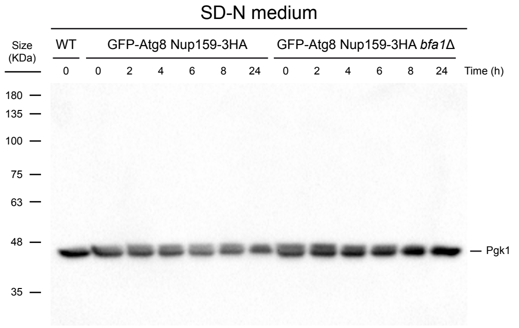

Raw images 5

Figure 6A: Original blot images

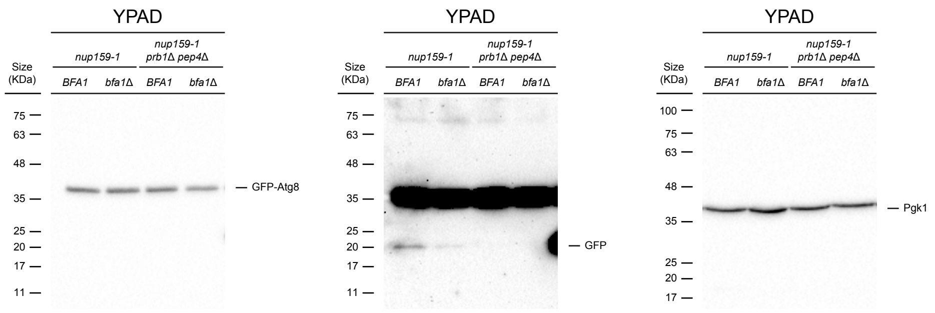

Figure 6C: Original blot images

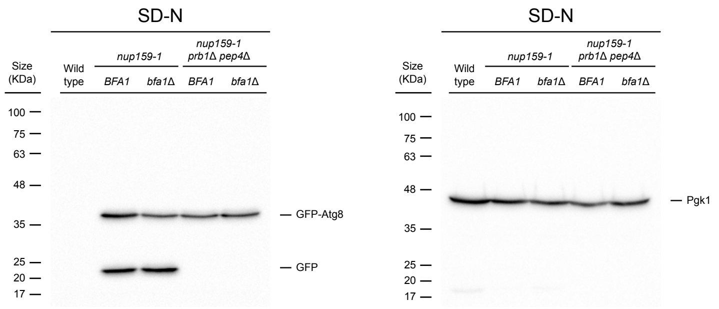

Figure S1A: Original blot images

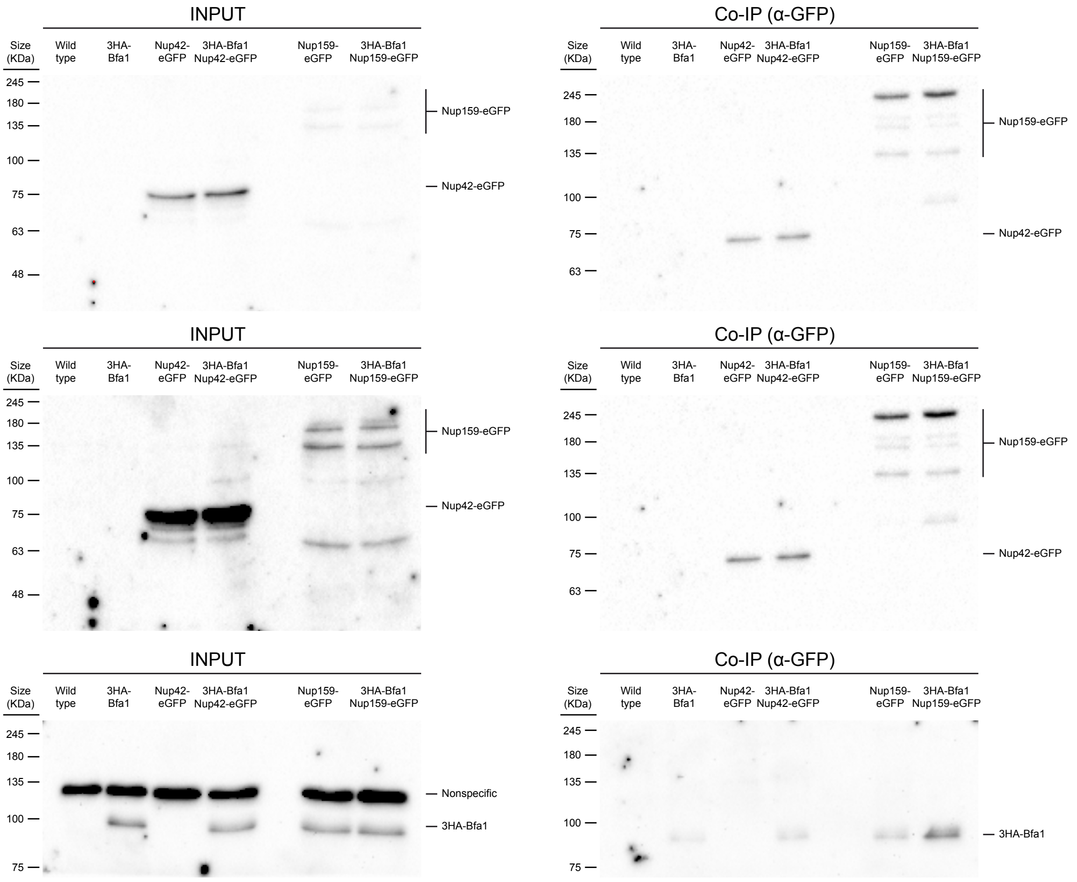

Figure S1B: Original blot images

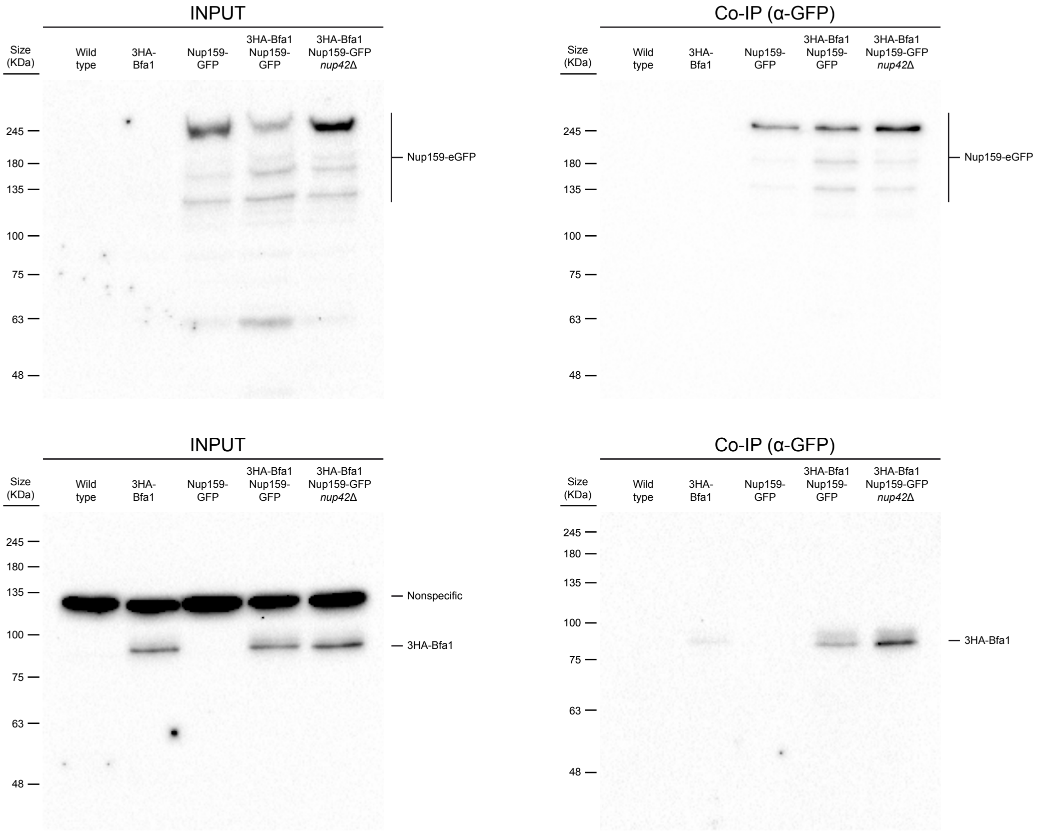

Raw images S1

Figure S2E: Original blot images

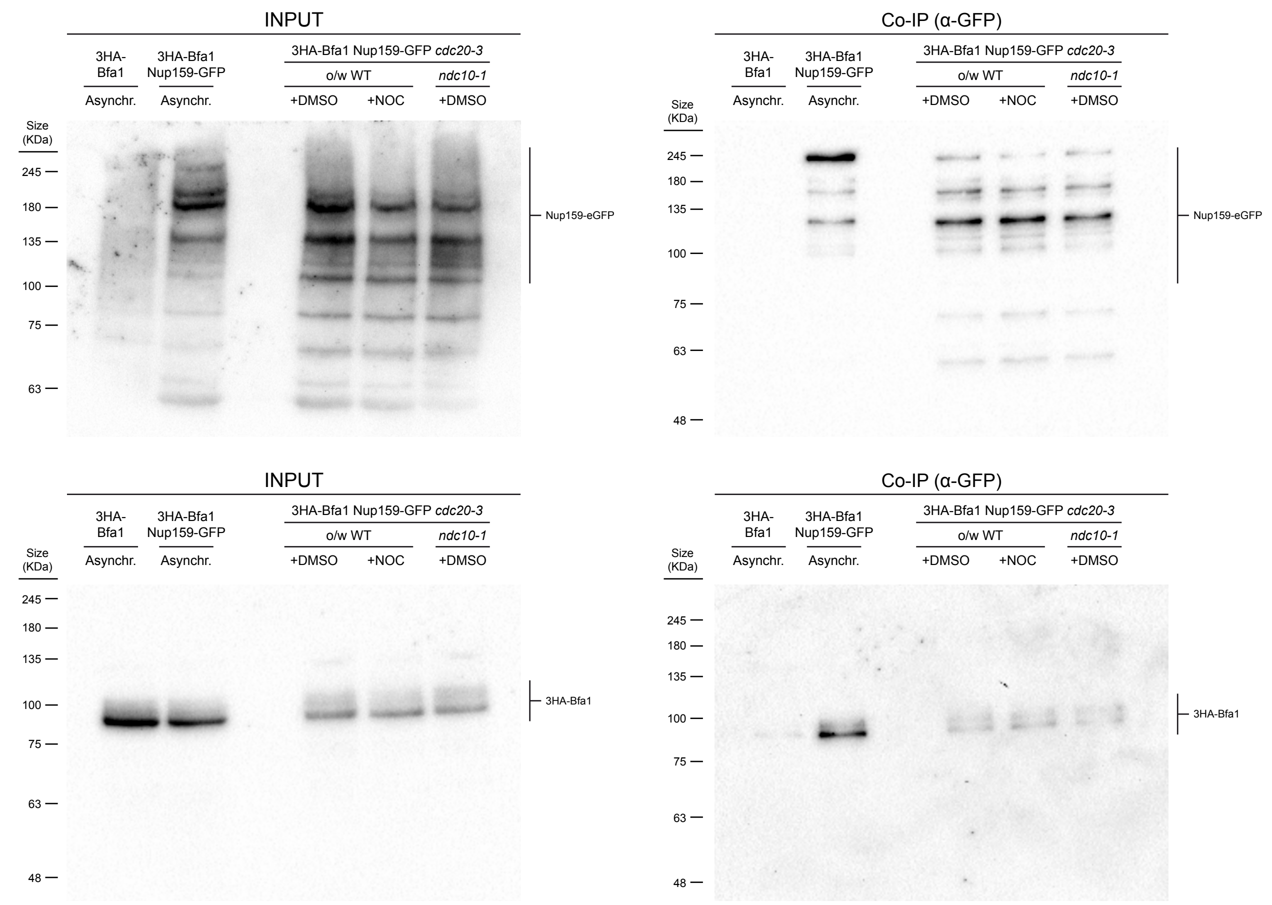

Figure S4A: Original blot images

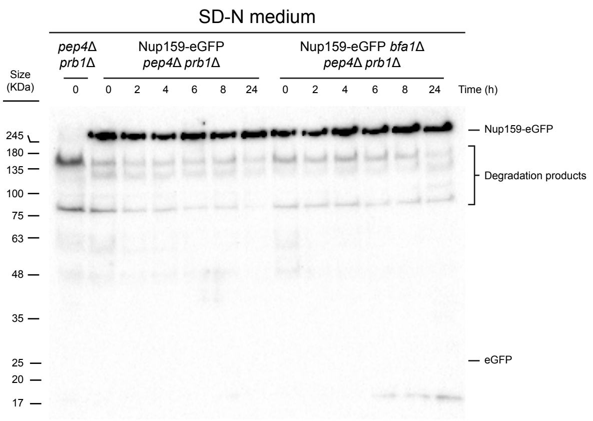

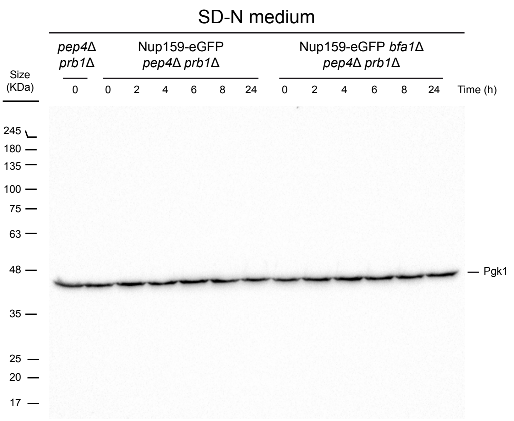

Figure S4B: Original blot images

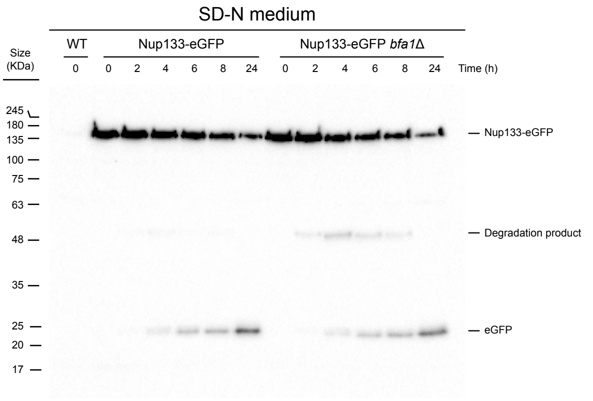

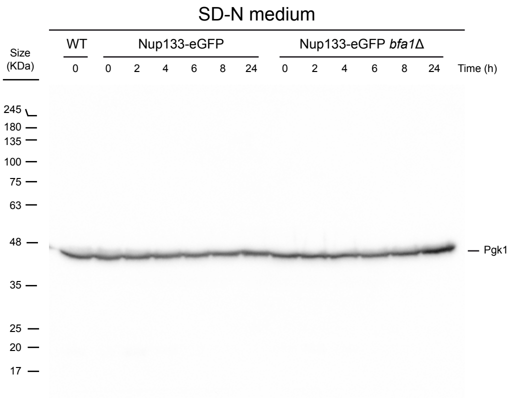

Figure S4C: Original blot images

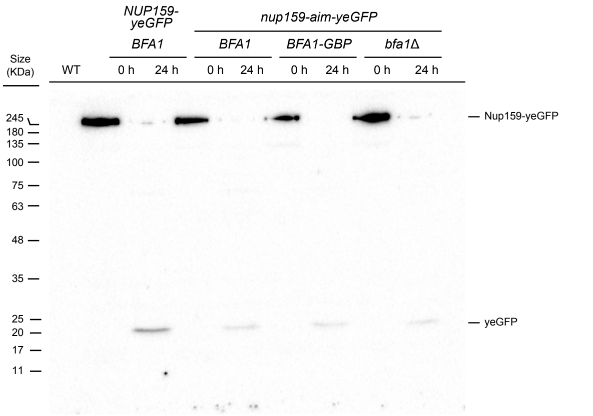

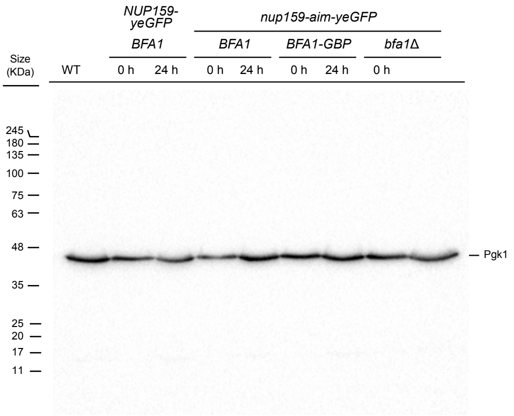

Figure S4F: Original blot images

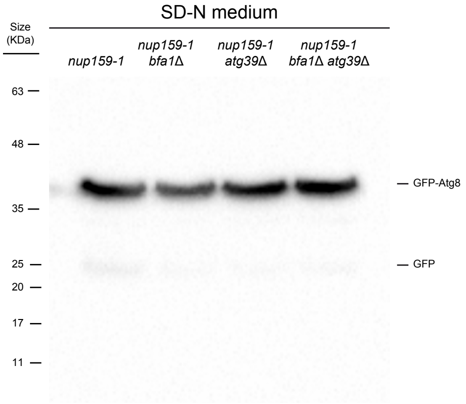

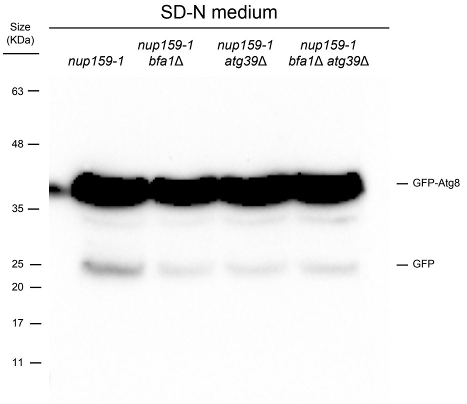

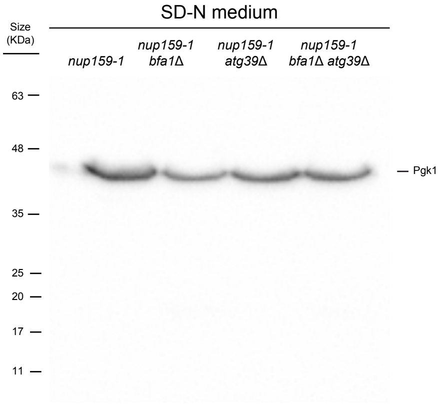

Raw images S4
