# Supplementary material for: Characterization of a novel interaction of the Nup159 nucleoporin with asymmetrically localized spindle pole body proteins and its link with autophagy
Source: PLoS Biol. 2023 Aug 3;21(8):e3002224. doi: 10.1371/journal.pbio.3002224 (PMC10437821; doi:10.1371/journal.pbio.3002224)
Supplement: Attachment — Submitted filename: Response to reviewers comments (PBIOLOGY-D-22-01108R1).pdf [file pbio.3002224.s011.pdf]

Reviewers' comments

We are grateful to all the reviewers for their comments and for their constructive suggestions, which helped us to reinforce our conclusions and to improve the overall quality of our original manuscript. Detailed herewith you can find a point-by-point answer to all the concerns raised.

Reviewer #1:

Overall, the investigation of the molecular interaction between Nup159 and Bfa1 is done thoroughly and detailed and can be convincing pending further technical improvements. The functional part, linking Bfa1 to autophagic degradation of NPCs is, however, underdeveloped and will require substantial further experimental work to support the authors claims.

We are thankful that the reviewer considered our study of interest for PLoS Biology. According to the reviewer's comments, in this revised version of our manuscript we have strengthened the characterization of the molecular interaction between Nup159 and Bfa1 and, more importantly, we have also greatly developed the functional link of its association with the process of autophagic degradation of NPCs. As a result, in our manuscript we now clearly demonstrate that Bfa1 and Nup159 collaborate in the selective autophagic clearance of misfolded or damaged nucleoporin complexes that contain Nup159 and that are recognized by the Atg8 factor. This specific pathway becomes highly relevant when damaged NPCs are the only trigger for autophagy, since its role can be taken over by alternative pathways when autophagy is globally induced in the cells. We hope that both the controls and the new experiments introduced finally contribute to more convincingly support our claims and to satisfy the reviewer's concerns.

<u>Major points:</u>

a) Nup159- Bfa1 interaction:

1 - Figure 1A-B: Comparison of different samples are done across different gels, which is not acceptable. Also, the manuscript would be strengthened by quantitative and not qualitative comparisons.

Figures 1A-B do not compare different samples across different gels. The reviewer might have got this impression because of the separation between samples in the gel in Figure 1A. In our initial co-immunoprecipitation experiments, due to the residual background signal that was sometimes observed in the control samples that only expressed 3HA-Bfa1, we intentionally separated each sample with an empty well in the gel. This allowed us to easily detect any residual transfer between samples and discard this as the potential origin of the background signal in the control lanes, which was instead caused by unspecific binding of 3HA-Bfa1 to the magnetic beads used in the assay. An explanatory note has been introduced in the legend to clarify this issue and, as a demonstration, we will include the original image of the whole gel in the raw data supplied with the manuscript. Finally, and following the reviewer's suggestion, we incorporated in the manuscript quantitative analyses of the protein levels in the Western blot experiments.

2 - Throughout the manuscript, the authors use different cell cycle mutants to induce arrests. They need to (a) provide data that cell cycle arrest worked as expected and (2) since cell cycle arrest often involves temperature shift also clearly indicate that control cells have been treated the same.

We did indeed check that the cell cycle arrest worked in each experiment and this have been clearly stated now in the manuscript. We only included this information in some (*e.g.*, Figure 5E,

Supplementary Figure 2A) but not all experiments, because in many instances the graphical representation does not provide any useful information (the results show 100% cells that arrest under the experimental conditions for all the strains analyzed) and, more importantly, because the experiments have their own internal control to evidence that the cell cycle arrest was successfully achieved. Specifically, since Bfa1 phosphorylation is dependent on the cell cycle, the efficiency of the arrest in the experiments can be demonstrated just by looking at the electrophoretic mobility of Bfa1 in the INPUT samples of the co-immunoprecipitation assays. In this way, Bfa1 is unphosphorylated in G1 and its phosphorylation increases as cells progress into the cell cycle, displaying a spread phosphorylation pattern in metaphase, and reaching its maximal phosphorylation level during anaphase (Valerio-Santiago *et al*., 2011). Finally, regarding the second concern raised by the reviewer, we have also clearly stated now that the control samples were always treated as the problem samples.

3 - Figure 3A: Input for Nup159 has a poor quality and should be repeated.

As requested by the reviewer, and after optimizing the conditions for the Western blot, we have reloaded the input for Nup159 in experiment 3A and included a new image, which improves the quality of the initial result.

4 - Figure 4B: I do not agree with the conclusion, since the authors also see less Nup159 in the input of *dyn2*Δ compared to the other cases.

As for the rest of experiments where comparisons are made, we have quantitatively estimated the amount of protein in Figure 4B to more strongly support our initial conclusion.

5- Figure 5: The interpretation of this "synthetic interaction experiment" is unclear and is not put very well in the context of the functional analysis in the next figures. Since currently, the authors do not have a loss-of-function/loss-of-interaction mutant, it may be advisable to leave these experiments out of the current paper and include in a future manuscript OR strengthen the link to the analysis of NPC autophagy.

Although we agree with the reviewer that a loss-of-interaction mutant would have been an extremely useful tool in order to further corroborate our results regarding the functional role of the Nup159-Bfa1 association, we do believe that the experiments forcing the Nup159-Bfa1 interaction with the GFP-GBP system are nonetheless informative, specially taking into account that their association is cell cycle regulated. In agreement with our data showing a decrease in the association of Nup159 and Bfa1 in metaphase (Figure 2), a forced interaction of both proteins perturbs proper spindle orientation specially during this cell cycle stage (Figure 4). Despite this is unrelated with the role of these proteins in autophagy later analyzed in the manuscript, this observation could provide an explanation for the reduced association of Nup159 and Bfa1 during metaphase. Furthermore, the fact that both Bfa1 and Nup159 (together with Dyn2) have a role in the process of spindle orientation, makes this an interesting and relevant result that we think that should be maintained. In contrast, we have removed the analysis in Figure 4C about the genetic interactions shown by *nup159-1*, *bfa1*Δ and *dyn2*Δ, since this particular experiment does not provide enough useful insight now and also because, with all the additional experiments included to more conclusively support a role of the Bfa1-Nup159 interaction in autophagy, it became necessary to limit the extension of the manuscript.

b) Role in Autophagic degradation of NPCs:

6 - Figure 6A-C: Why are Nups completely degraded after 2 h SD-N treatment? This is in contrast to previous studies and should be discussed. Importantly, the phenotype in the bfa1-mutant is

very mild and not apparent after 24h. Given this, individual replicates of experiments need to be shown both as qualitative western blots and in the quantification, where mean +- SD is not acceptable any longer. Also, conclusions need to be toned down given the mild effect. In the same vein, Supplement Figure 3B shows actually no difference in the degradation of nup133 +/- Bfa1. This is somehow unexpected since NPCphagy should degrade Nup133 as well.

Although the Nup159 protein is indeed rapidly degraded once cells are deprived from nitrogen, we do not agree with a more general statement regarding our autophagy experiments under these conditions. In this way, in our analyses, not all nucleoporins were soon degraded after cells were transferred to SD-N and, overall, our results do not greatly differ from previous studies. Instead, we believe that the fast reduction in protein levels after cells were deprived of nitrogen is rather specific of Nup159. This nucleoporin, as originally pointed out in our manuscript, is particularly prone to degradation in protein extracts. In contrast, Nup133 is more stable under the same conditions (Supplementary Figure 3B). The same is also true in our hands for Nup42, Nup116 or Nup192 (our unpublished observations). Accordingly, different nucleoporins have been shown to be degraded with distinctive kinetics depending on their location within the nuclear pore, which could be one of the reasons that explain the rapid autophagic clearance of Nup159, a protein located in the outer nuclear pore surface. Furthermore, there are in fact many examples in the bibliography that corroborate that Nup159 is indeed an unstable protein that is more efficiently degraded than other nucleoporins after nitrogen starvation (e.g., Lee *et al.*, 2020 (see Fig. 2A), Tomioka *et al.*, 2022 (see Figs. 1B, S1B, S2E)). We believe that the rapid degradation of Nup159 also explains why, in most articles, only the initial and final time points are usually displayed for this nucleoporin instead of complete time courses showing protein levels after cells are transferred to nitrogen-depleted conditions. It is important to highlight, in any case, that the unstable nature of Nup159 does definitely not arise from our experimental setup, since not only it is not observed with other nucleoporins, as previously indicated, but more importantly it does not take place in a *prb1*Δ *pep4*Δ mutant.

Regarding the second concern, the reviewer is right in that the phenotype shown by the *bfa1*Δ mutant is very mild under nitrogen starvation. However, as extensively developed in our response to major point #2 from reviewer #2, this result was not completely unexpected based on the fact that *(i)* growth under nitrogen starvation conditions causes an overall induction of many autophagy processes and that *(ii)* the autophagic clearance of NPCs is not exclusively carried out in a Nup159-dependent manner, but it can also occur by other mechanisms such as Atg39-mediated nucleophagy or piecemeal microautophagy (Yin *et al.*, 2020). Indeed, in our revised manuscript, we now demonstrate that the combined role of Nup159 and Bfa1 can be taken over by alternative pathways when autophagy is globally induced in the cells, but that this degradation pathway becomes highly important when damaged NPCs are the only trigger for autophagy. We did not introduced replicates of the experiments of Nup159-GFP degradation in medium without nitrogen because we provided the graph with the average and thus considered it redundant. Furthermore, now that we more conclusively demonstrate using different experimental approaches that the autophagy defect is undoubtedly observed when NPC damage is specifically induced, we believe that it unnecessarily increases the amount of data. However, we are attaching in our rebuttal a figure showing the requested replicates and we will submit it with the raw data if considered necessary (Figure for reviewers 1).

Finally, and regarding the autophagic degradation of Nup133, we also believe that it is less efficient in the absence of Bfa1 than in the wild type strain, as evidenced by the accumulation of intermediate GFP-degradation products in *bfa1*Δ cells, even though the effect is indeed not as evident. Here, it is also important to stress that Nup133 is a nucleoprotein from the inner core region of the NPC and thus that its autophagic turnover is more limited. Furthermore, even if Nup133 degradation were not affected, this result would not discard a specific role of Bfa1 in

autophagy together with Nup159, since there are contradictory results in the bibliography regarding whether Nup159 mediates only the selective clearance of specific Nups or whether it acts as a receptor that mediates autophagic degradation of the whole nuclear pore (reviewed in Yin and Klionksky, 2020). Notably, our new results included in the revised version of the manuscript indicate that, if not the whole NPC, it must be nucleoporin subcomplexes that are targeted to autophagy, since Nup192-yEGFP foci are also less accumulated in the vacuole of *atg15*Δ cells in the absence of *BFA1* (Supplementary Figures 4A and B).

7 - I suggest that the authors should also investigate Nup degradation under the same conditions they see the strongest interaction between Bfa1 and Nup159. Moreover, they should test whether the nup159-AIM mutant has a similar effect.

Following the reviewer's suggestion, we have analyzed the role of Bfa1 in the autophagic clearance of nucleoporins in cells that express the *nup159-AIM* allele, and also checked the effect that a forced Nup159-Bfa1 interaction has in this process. According to previous observations, degradation of the Nup159 nucleoporin was impaired in *nup159-AIM* mutant cells (Supplementary Figures 3C and 3D), as a consequence of the reduced interaction of nup159-AIM with the autophagy receptor Atg8 (Lee *et al*., 2020). Remarkably, the lack of Bfa1 did not further enhance this defect in the autophagic degradation of nup159-AIM (Supplementary Figures 3C and 3D). Likewise, a forced interaction between nup159-AIM and Bfa1 using the GFP-GBP strategy was not able to rescue the defect of the AIM mutation (Supplementary Figures 3C and 3D). These are very interesting results that support our idea that Bfa1 likely acts at the very initial steps of Nup159-dependent autophagy, likely acting as an adaptor that facilitates the recognition of Nup159 by the autophagic machinery. Our results also suggest that, despite the epistatic role of Bfa1 in the Atg8-dependent pathway, this adaptor role of Bfa1 cannot, however, correct the defect in the association of nup159-AIM to Atg8.

8 - Interpretation of Figure 6D is unclear. Who says that these foci are autophagic bodies / autophagosomes. I suggest to do the same assay in atg15Δ. Moreover, one could delete Atg8,1,7 or 5 to see if the foci disappear.

Following the reviewer's suggestion, we have confirmed that the Nup159-yEGFP foci originally shown in Figure 6D (now Figure 6F) are not observed in an *atg8*Δ mutant background (Supplementary Figure 4F). Consequently, these foci do actually represent useful markers for autophagic bodies / autophagosomes. In any case, and to more convincingly demonstrate a role of the Bfa1-Nup159 association in autophagy, we have strengthened our conclusions by complementing our initial results following different experimental approaches. First of all, we used the same methodology described by Lee *et al*. to analyze the role of Nup159 in autophagy (Lee *et al*., 2020). Specifically, they followed the delivery of nucleoporins to autophagosomes after cells were deprived of nitrogen by tagging Nup192 with eGFP and estimating the percentage of cells with vacuolar Nup192–eGFP foci in an *atg15*Δ mutant background, which lacks a lipase that is indispensable for dissolving autophagosomal membranes in the vacuole, thereby facilitating visualization of NPC degradation intermediates (Lee *et al*., 2020). By using the same approach, not only we corroborated the increase in vacuolar Nup192-yEGFP foci in *atg15*Δ cells starved of nitrogen, but we further demonstrated that autophagosomes are significantly less accumulated in vacuoles in the *atg15*Δ *bfa1*Δ mutant, in agreement with a role for Bfa1 during the autophagic clearance of nucleoporin complexes (Supplementary Figures 4A-B). Next, in a second strategy to reinforce our data, we followed the delivery of membrane-embedded nucleoporins to autophagosomes as a consequence of the specific accumulation of damaged nuclear pores in *nup159-1* cells by analyzing the distribution of the Atg8 core autophagic factor tagged with GFP. According again with our hypothesis, GFP-Atg8 foci formed as a consequence of *nup159-1* expression were accumulated in the vacuole of *atg15*Δ cells, but

the presence of GFP-Atg8 in vacuolar autophagosomes was reduced as a consequence of the lack of Bfa1 (Figures 6D and 6E). Finally, we evaluated the effect of deleting *BFA1* in cells lacking the Rab family GTPase Ypt7. Since Ypt7 is necessary for the fusion of the autophagosome with the vacuoles, the *ypt7*Δ mutant is also experimentally used to increase the number of observable autophagosomes (Torggler *et al*., 2017). Using this approach, our results show that, while *ypt7*Δ cells more frequently accumulated extranuclear foci of both Nup192-yEGFP and Nup159-yEGFP in the periphery of the vacuole, the number of these foci was significantly reduced in the absence of Bfa1 (Supplementary Figures 4B and 4C-E).

<u>Additional points:</u>

9 - They started with a Y2H screen but none of the data are available. They should provide at least the data for the case they report. In general, I would suggest to publish the results of the whole yeast-two hybrid screen.

We appreciate the reviewer's comment but, since in our manuscript we exclusively focus in describing the interaction between the nucleoporins and Bfa1, which we demonstrate by different approaches and functionally characterize, we do not think it is necessary to detail the results from the whole two-hybrid screen. This screen was only used as a starting point for our research, providing us with the initial observation that unveils the role of Bfa1 in the autophagic degradation of the nucleoporins, and we would rather not disclose other interactions that we could also characterize in the future.

10 - For all western blots size markers are not displayed throughout the manuscript.

We did not include size markers to not complicate too much the figures displaying Co-IPs, since a lot of information was already included. We will, however, facilitate a merged photo of the gel with a colorimetric image showing the molecular weight marker. Also, we included in this rebuttal letter a figure displaying the different sizes for the analyzed proteins (Figure for reviewers 2).

11 - Labelling of the IP data is not very clear. It should be indicated in the figure against which tag the IP was performed.

Labelling of the IP data has been modified and it is hopefully now clearer to interpret.

12 - The statement "The GFP-GBP methodology has the additional advantage that allows evaluation of the localization of the protein complex by fluorescence microscopy." Is misleading since the GFP-GBP methodology works by forcing two proteins together bringing with it artificial localization of the complex.

The statement has been changed, as requested.

Reviewer #2:

Overall, the data are heavily reliant on co-immunoprecipitations/Western blots that vary in quality (e.g. relative amounts/stability of proteins in western blots are inconsistent between experiments) and it remains unknown whether the interaction between Nup159 and Bfa1 is actually direct. Furthermore, there is no clear sense of what the ultimate function of the interaction is as there are no experiments that specifically disrupt it. There is, however, an

attempt to force the interaction by using a GBP-GFP approach but these data are difficult to interpret.

We understand the reviewer's concerns and appreciate the suggestions provided to improve our initial manuscript. We have followed the reviewer's recommendations and also introduced a significant number of novel experiments to strengthen our initial conclusions. We believe that the new data incorporated in the revised version of our manuscript provides enough experimental support to more irrefutably confirm the interaction between Nup159 and Bfa1 and, more importantly, its functional relevance. In this sense, we now exhaustively characterize the function of the Nup159-Bfa1 association and clearly demonstrate that their interaction is specifically relevant for the clearance of misfolded or damaged nucleoporin complexes that contain Nup159 and are recognized by the autophagy factor Atg8

Thoughts for the authors should they wish to improve technical aspects of the paper:

1) Many of the Western blots presented in this manuscript are of inconsistent quality and lack quantification. For example, the amount of 3HA-Bfa1 that was co-IP'ed with Nup159-GFP in Figure 1A looks to be much more than the amount in Figure 2A, although these should be identical experiments. In addition, although the authors claim that the interaction between Bfa1 and Nup159 is reduced in metaphase, the amount of Bfa1 that co-IP'd with Nup159-GFP in Figure 3B looks to be similar to the amount in 2A from asynchronous and G1 arrested cells. There is also no way to assess the efficiency of the IP without knowing the fraction of the input and bound fractions loaded on the gels.

The Western blots have been quantified and, in some instances, the whole experiment was repeated (Figure 2A, Figure 2B) or samples were reloaded (Figure 3A) to get images of a higher quality, as requested by both reviewers (see also the response to Reviewer 1 comments). Regarding the differences among figures, it is important to highlight that they correspond to independent experiments and that different gel exposures were used in each case to better visualize the changes in the association. Although we believe that the results are extremely consistent, in the revised version we have nonetheless tried to use more similar exposures to avoid possible confusions. Finally, it is worth noting that quantifications and comparisons within an experiment and between different figures are complicated by the unstable nature of the Nup159 protein, which also introduces some variability.

2) The idea that the Bfa1-Nup159 interaction promotes the degradation of NPCs by autophagy is not well supported by the data and is open to alternative explanations. It is also conceptually challenging how an interaction at the SPB could lead to degradation of NPCs. In terms of the presented data, the authors suggest that degradation of Nup159 (and Nup133) is delayed in the absence of Bfa1. This might be the case but the upstream signaling driving autophagy would have to be more thoroughly explored as there does not appear to be any major change in the kinetics of degradation per se. Indeed, based on their blot in Figure 6A, the degradation kinetics of Nup159 look identical with or without Bfa1. According to their quantification of the relative amount of free GFP in Figure 6C, it seems like there could be slightly less degradation at 6 hours of nitrogen starvation in *bfa1Δ* cells compared to wildtype, however, comparing this to the relative amount of full-length protein from Figure 6B (which is also reduced compared to wildtype), it is unclear if there would be any evidence of reduced degradation if the free GFP band was normalized to the full length Nup159-GFP band (as is the standard method of quantifying autophagic degradation).

An increasing body of evidences demonstrate that the SPBs and the NPCs collaborate in the regulation of key cellular processes and even share common components. Hence, we do not

consider conceptually challenging that an interaction between components of both structures could mediate the autophagic degradation of nucleoporins. Furthermore, NPCs are not randomly distributed in *S. cerevisiae*, but organized in clusters that concentrate around the SPBs (Winey *et al.*, 1997). This observation provides a plausible functional significance for the need of such an interaction, which might contribute to prevent the inheritance of dysfunctional NPCs in the daughter cell based on the asymmetric loading of Bfa1 on the SPB that enters the bud in anaphase. On the other hand, the modest defect in the autophagic degradation of Nup159 and Nup133 that the reviewer points out was not completely unexpected taking into account that, as indicated in our original manuscript, *(i)* growth under nitrogen starvation conditions causes an overall induction of many autophagy processes and *(ii)* the autophagic clearance of NPCs is not exclusively carried out in a Nup159-dependent manner, but it can also occur by other mechanisms such as Atg39-mediated nucleophagy or piecemeal microautophagy (Yin *et al.*, 2020). In the revised version of the manuscript, we now more conclusively demonstrate that the Nup159-Bfa1 interaction is specifically relevant for the Nup159-dependent pathway of autophagic degradation of nucleoporins, but not for the overall induction of autophagy in the cell, and that their association collaborates with other autophagic pathways for an efficient recycling of the NPC components. In this sense, the viability of cells treated with rapamycin becomes highly dependent on Bfa1 when they further accumulate damaged NPCs as a consequence of the expression of the *nup159-1* allele, even at the permissive temperature (Figure 5E). Notably, this viability defect of *nup159-1 bfa1*Δ cells after rapamycin treatment was similar to that shown by a *nup159-1 atg39*Δ mutant (Figure 5F). Furthermore, in contrast to cells growing under nitrogen-deprived conditions (Figure 5D), GFP-Atg8 degradation was less efficient in cells lacking Bfa1 after autophagy was selectively triggered by nuclear pore damage caused by the expression of the *nup159-1* allele (Figures 6A-C). These results are in agreement with our idea that Bfa1 collaborates in the initial steps of Nup159-dependent autophagy, probably facilitating the direct recognition of damaged Nup159-containing nucleoporin complexes by the autophagic machinery, and not acting further upstream in the autophagy signaling pathway. Finally, as extensively explained in our response to reviewer #1 (Major point #8), we have used different experimental approaches to undoubtedly demonstrate, by following the delivery of autophagosomes, that the process of autophagic clearance of nucleoporin subcomplexes is specifically affected in the absence of Bfa1 (Supplementary Figure 4).

The aforementioned results can explain the somewhat subtle defect in the autophagic clearance of the nucleoporin. In any case, as a final note regarding the kinetics of Nup159 degradation in nitrogen-deprived medium, we would further like to clarify that although the initial levels of Nup159 were indeed higher in the *bfa1*Δ mutant, not only we observed a reduced degradation of this nucleoporin during the initial stages of autophagy, but we did also evidence a decrease in its degradation rate when absolute Nup159-yEGFP/free yEGFP levels were normalized to the initial time point (time = 0 h), as pointed out by the reviewer.

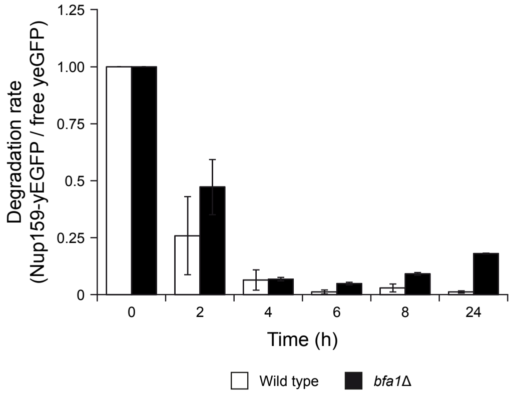

3) The bimolecular fluorescence complementation experiment in Figure 1C could be supported by additional specificity controls (e.g. additional nups and SPB components) and perhaps the incorporation of the temperature sensitive mutants that are thought to prevent the interaction (metaphase arrested) or promote the interaction (anaphase arrested).

Both Bfa1-VC and Nup159-VN have successfully been used in prior BiFC assays and shown to be functional (our own results and Adams and Wente, 2020). In this sense, we have previously demonstrated in our lab that Bfa1-VC shows a strong BiFC interaction with Bub2-VN, another SPB component that interacts with Bfa1 and thus serves as a positive control.

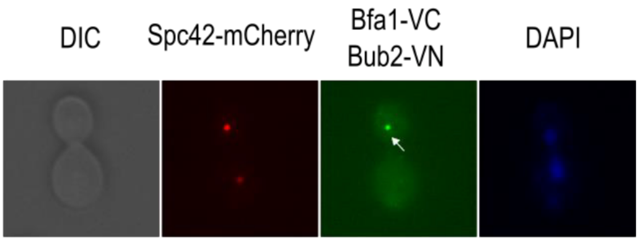

In any case, as requested by the reviewer, we have included additional specificity controls for our BiFC experiments to more strongly support our claims. As such, in our revised manuscript we demonstrate that, interestingly, Bfa1-VC also interacted in BiFC experiments with Dyn2, another nucleoporin from the Nup82 complex. Bfa1 further displayed a very limited association with Nup100, which directly interacts with Nup159-Nup82 (Yoshida *et al*., 2011). However, Bfa1-VC did not interact with Gle1 or Nup42, which are nucleoporins that localize to the cytoplasmic side of the NPCs. It is important to highlight that, in our analyses, Nup42 did not co-immuno-precipitate with Bfa1 either, further supporting the specificity of the interactions observed.

Finally, following the reviewer's suggestions, we have evaluated the BiFC interaction in different temperature sensitive mutants that facilitate either a metaphase (*cdc13-1* and *cdc20-3*) or an anaphase (*cdc15-2*) arrest when grown at the restrictive temperature. In agreement with our co-immunoprecipitation experiments, the BiFC interaction between Nup159 and Bfa1 is reduced in the metaphase-arrested cells, while it is favored when cells are blocked in anaphase (Figure 2A). This result not only confirms the validity of the BiFC assays, but constitutes a further demonstration that the Nup159-Bfa1 association is cell cycle-regulated.

4) Please include blots to show the degradation of Cdc20-AID and Kar9 in the presence of IAA.

We did not check the degradation of Cdc20-AID or Kar9-AID since these alleles have been extensively characterized and thus was considered not necessary. Instead, as previously indicated, we checked that the cells clearly displayed the expected phenotype after auxin addition. Specifically, degradation of Cdc20-AID was confirmed by the strong arrest of the cells in metaphase, as shown by the analysis of spindle and nuclear morphology and the phosphorylation pattern of Bfa1 in the INPUT samples (Figure 2B), while Kar9-AID depletion was evidenced based on the increase in mispositioned spindles (Figure 3C).

5) Do cells in Figure 3 B-D include the Kar9-AID-9Myc construct and dyn1Δ as indicated from the call out in the text? If so, please fix. If not, it is unclear what the IAA drug is doing.

The cells indeed included both the Kar9-AID-9Myc construct and *dyn1*Δ, but this was not correctly indicated in the Figure legend nor in the strain list. We apologize for this mistake, which has been fixed as requested.

6) Please include genotypes of the cells shown in Figure 3B.

The genotype of the cells is now specifically indicated in Supplementary Table 1, as requested.

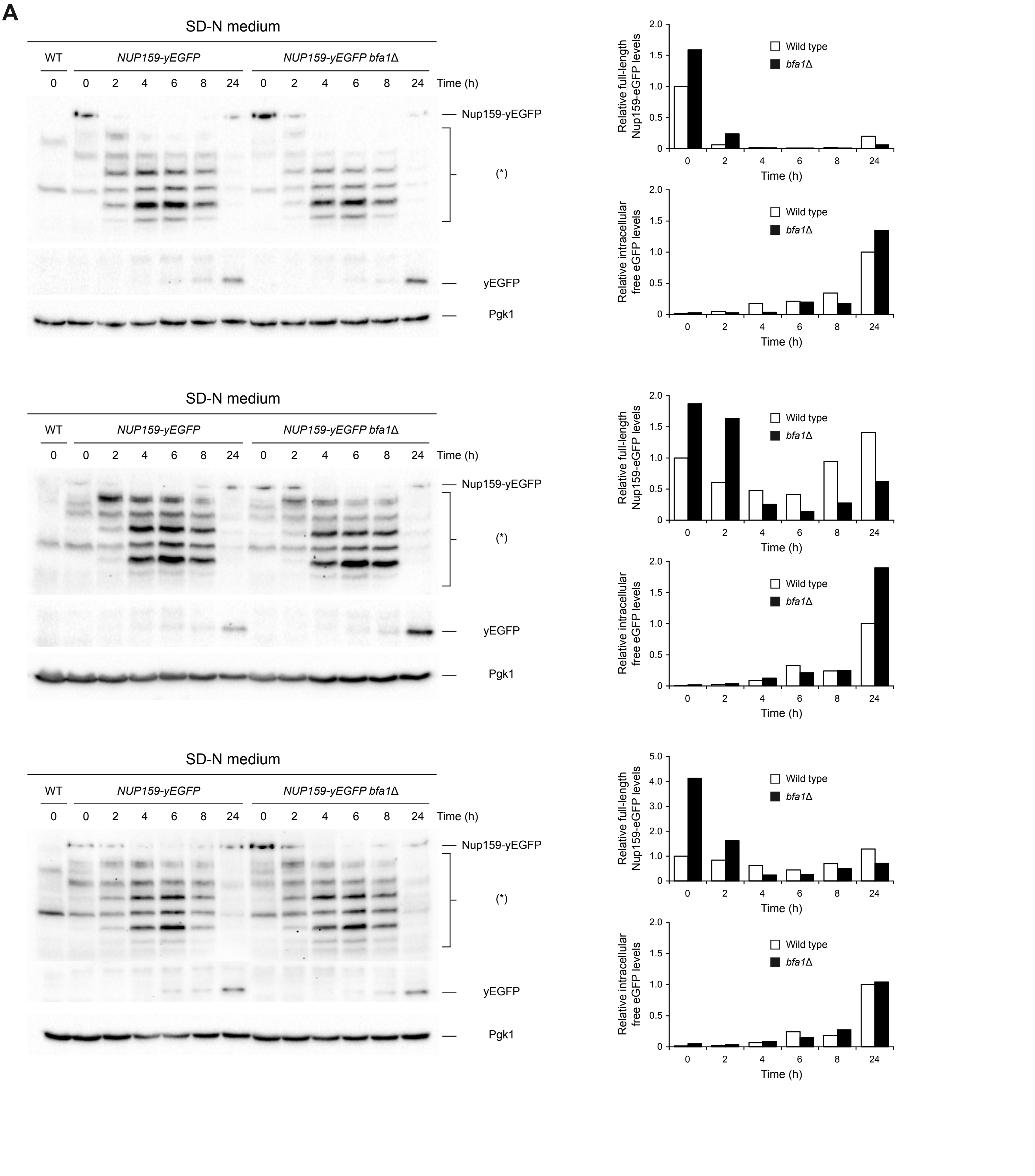

Figure for reviewers 1

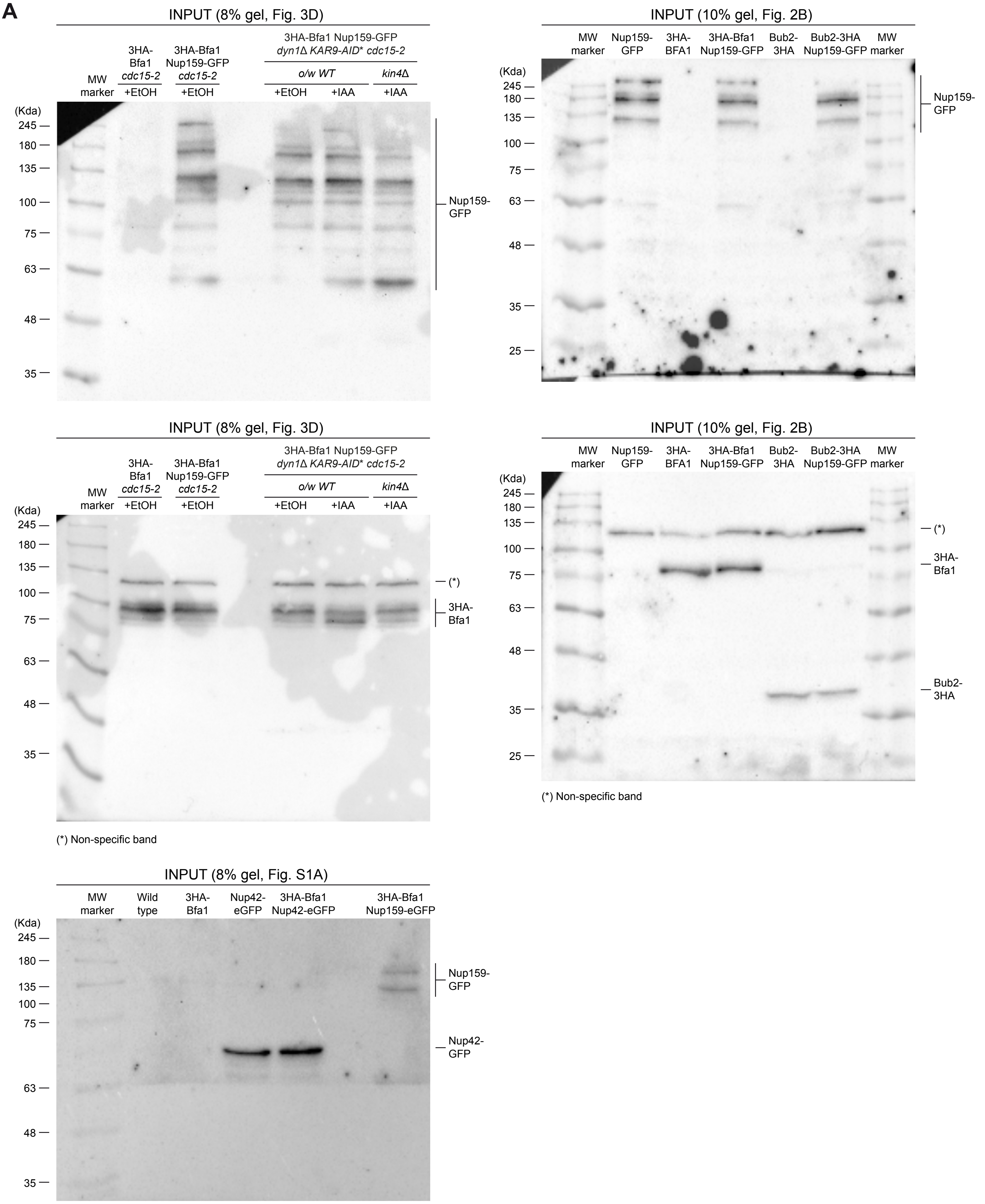

Figure for reviewers 2
