# Supplementary material for: Characterization of a novel interaction of the Nup159 nucleoporin with asymmetrically localized spindle pole body proteins and its link with autophagy
Source: PLoS Biol. 2023 Aug 3;21(8):e3002224. doi: 10.1371/journal.pbio.3002224 (PMC10437821; doi:10.1371/journal.pbio.3002224)
Supplement: Attachment — Submitted filename: Response to reviewers comments (PBIOLOGY-D-22-01108R2).pdf [file pbio.3002224.s012.pdf]

Reviewers' comments

We are grateful again to both reviewers for their comments after the initial revision of our manuscript. Detailed herewith you can find a point-by-point answer to all final concerns raised.

Reviewer #1:

Overall, I find the revised version of the paper improved in some parts and I think the investigation on the molecular interactions is worth to be published after including necessary controls. However, I am not convinced that the data allows to link these interactions with NPC quality control. Therefore, this claim/interpretation should be removed from the paper.

We are thankful to the reviewer both for recognizing the improvement of our manuscript after the initial revision and, particularly, for considering our investigations worth to be published. Together with the thorough characterization of the molecular interaction between Bfa1 and Nup159, we followed two different approaches to demonstrate the participation of Bfa1 in the Nup159-dependent autophagy pathway:
1.- By globally inducing autophagy in nitrogen-deprived media, and then specifically focusing on the targeting of Nup159-GFP into autophagosomes towards the vacuole.
2.- By selectively inducing nuclear pore damage using the *nup159-1* allele and then evaluating the efficiency of the autophagy process both by following GFP-Atg8 delivery to autophagosomes and also its degradation in the vacuoles.
We appreciate the reviewer's concerns regarding a specific function of both proteins in NPC quality control. Hence, as further explained in our response to reviewer #2, we have eliminated this particular statement and also toned down some of our prior conclusions, which we only discuss as possible hypotheses that will be worth exploring in the future. Nonetheless, we firmly believe that our results combining both described strategies clearly demonstrate a collaborative role of Bfa1 in Nup159-mediated autophagy, which is what we now principally endorse in our manuscript. In agreement with these changes, and following a suggestion by Reviewer #2, we have finally also subsequently modified the title of our manuscript to be more circumspect to the main conclusions that are finally established.

Besides the previous general considerations, we have also addressed all other final points raised by the reviewer:

Major points:

a) Nup159- Bfa1 interaction:

1 - Figure 1 - Please support your argument in the rebuttal letter by showing full images of western blots. Also, please include Molecular Weight markers.

We have incorporated full images of Western blots, including the corresponding molecular weight marker, into the supplementary figures (new Figures S1A and S1D).

2 - Figure S1a - How can you conclude specificity with two GFP-tagged proteins?

The co-immunoprecipitation of 3HA-Bfa1 with Nup42-eGFP was carried out using a different strain (F3238) than that used to co-immunoprecipitate 3HA-Bfa1 with Nup159-eGFP (F3294), as originally noted in the list of strains included in our manuscript (Table S1). Therefore, specificity can be conclusively established by comparing each strain with the corresponding controls

carrying either 3HA-Bfa1 or the untagged nucleoprotein. In any case, to avoid confusion, we have now further clarified this in the figure legend.

3 - Figure 1B - "immunoprecipitation assays, which indicates that this nucleoporin is able to interact with both components of the GAP complex (Figure 1B)." This statement is wrong, rephrase "Nup159 can interact with the GAP complex but not necessarily directly interact with both components.".

The statement has been rephrased, as indicated.

4 - Figure 1C - include grey scale image for the single channels. PhC + merged can be removed since there is no additional information gained from it.

We have included grey scale images for the singles channels of Figure 1C in the supplementary data (Figure S1E).

5 - Cell cycle regulation - Yes, cell cycle arrests in budding yeast are often more efficient compared to other systems. The authors argument that they worked to 100% in all strains in all experiments is however hard to believe. Please include the according quantifications in the manuscript. There is enough room in the supplementary data.

As requested by the reviewer, we have incorporated the quantifications for the cell cycle arrests into the supplementary data (new Figures S2A-C and S2F).

6 - "Interestingly, Bfa1-VC also showed positive BiFC interaction with Dyn2-VN, another nucleoporin from the Nup82 complex" Dyn2 is not a nucleoporin, rephrase to "component"

We have changed the text, as indicated.

7 - Figure 4A - Co-IPs are difficult to evaluate if bait is not pulled down to equal amounts. The binding partner may already be in saturation.

Although the increased association of Bfa1 with the truncated Nup159-1 protein is later used as possible evidence that the Nup159-Bfa1 interaction might be favored when nucleoporin aggregates accumulate, the aim of the experiment in Figure 4A was only to demonstrate that the C-terminal domain of Nup159 is not required to establish the interaction of this nucleoporin with Bfa1. As noted by the reviewer, in these experiments the Nup159-1 mutant protein seems to be consistently less efficiently co-immunoprecipitated than the wild type nucleoporin. While we understand the reviewer concern, we did not find any indication that the protein levels of any of the binding partners might be limiting in our experiments. Incidentally, according to published data (Ghaemmaghami *et al*., 2003), it is estimated that there are 1230 Nup159 molecules and 1380 Bfa1 molecules per cell. Thus, Nup159 and Bfa1 are present at similar amounts in the cells and, in principle, the global levels of both binding partners do not seem to suggest that they could be in saturation with respect to each other. In any case, to acknowledge the issue raised by the reviewer, we have introduced this possibility in the text.

9 - Figure 5B,C - Include statistics / biological replication to show significance.

The biological replicates have been included into a supplementary figure to show significance (new Figure S3).

b) Role in Autophagic degradation of NPCs:

10 - Figure 6A-C: The main conclusion of this part of the paper are not backed up by experiments. An alternative explanation may be an mRNA export defect of *nup159-1*. Induction of autophagy needs, however, increased expression of Atg8 (as it is depleted by degradation). This hypothesis is sufficient to explain all effects observed in this part of the paper and is not refuted by any of the data.

Regrettably, the fact that the *nup159-1* allele might display mRNA export defects is inherent to the role of the Nup159 nucleoporin in facilitating the transport of RNA molecules. Such a caveat is in fact extensive to other mutant alleles coding for different nucleoporins that have been shown to play moonlighting roles in the cell. Nonetheless, we consider that the hypothesis of our observations being a consequence of mRNA export defects can be ruled out by some of the results shown and that our main conclusions are satisfactorily sustained by the experiments in our manuscript. In this sense, it is important to highlight that:
a) Although we have found that the *nup159-1* mutant is an excellent model to selectively induce nuclear pore damage and, therefore, to more specifically evaluate the role of Bfa1 in Nup159-mediated autophagy, we additionally confirmed the role of Bfa1 in these processes by analyzing clearance of Nup159-GFP and Nup192-GFP, as well as their targeting to autophagosomes, after autophagy was instead globally induced in otherwise wild type cells by nitrogen depravation (Figures 6F-I and Supplementary Figures S5A-F).
b) Even if *nup159-1* displayed mRNA export defects, there is an evident difference in the ability of *nup159-1* cells expressing Bfa1 and the *nup159-1 bfa1*Δ mutant to efficiently degrade GFP-Atg8 and to target this autophagic factor to vacuoles. These processes are undoubtedly impaired in the absence of Bfa1, despite both strains carrying the *nup159-1* allele (Figures 6A-E).
c) Since C-terminal tagging of Atg8 renders this protein not functional, for our experiments we used a strain in which Atg8 is N-terminally tagged with GFP and expressed from the *CUP1* gene promoter. This strain was generated from a plasmid originally designed in Daniel J. Klionsky's lab (Nair *et al.*, 2012) and widely used afterwards by many different laboratories as an essential tool to analyze autophagy. This system ensures a continuous expression of GFP-Atg8 throughout the experiment to more easily evidence autophagic processes. Furthermore, if necessary (not in our case), this construction also allows to overexpress GFP-Atg8 by adding cupper to the medium, thereby facilitating its visualization. As a consequence, we do not expect changes in *ATG8* global levels after autophagy is induced in this genetic background. Accordingly, in our experiments, total levels of GFP-Atg8 are similar in *nup159-1* cells in the absence or the presence of Bfa1 (Figure 6A). It is important to note, nonetheless, that this further supports that the reduced levels of free GFP in *bfa1*Δ cells are indeed the result of a less efficient autophagic degradation of GFP-Atg8 when nucleoporin aggregates accumulate in cells that lack Bfa1.
d) In any case, to circumvent the issue described in the prior point, and to demonstrate that the *nup159-1* allele does induce the activation of autophagy, we have measured *ATG8* expression by quantitative RT-PCR in cells that exclusively express the endogenous *ATG8* gene. As shown in an accompanying figure (Figure for Reviewers A), and as anticipated by the reviewer, *ATG8* expression is similar in wild type and *bfa1*Δ cells but it is increased in *nup159-1* cells, supporting that autophagy is indeed triggered as a consequence of the expression of the mutant nucleoporin.

11 - Figure S3b - The effect on Nup133 levels is very minor at best.

As already indicated in our previous rebuttal letter, we agree in that the effect of the lack of Bfa1 in Nup133 degradation after autophagy is globally induced by nitrogen depravation is minor (new Supplementary Figure S4B). However, as also discussed in our response, it is important to stress that Nup133 is a nucleoprotein from the inner core region of the NPC and, hence, that its

autophagic turnover is expected to be more limited. Furthermore, even if Nup133 degradation were not affected, this result would not discard a specific role of Bfa1 in autophagy together with Nup159, since there are contradictory results in the bibliography regarding as to whether Nup159 mediates only the selective clearance of specific Nups or it acts as a receptor that facilitates the autophagic degradation of the whole nuclear pore (reviewed in Yin and Klionsky, 2020). We, in any case, favor the idea that, if not whole NPCs, it must be at least nucleoporin subcomplexes that are targeted to autophagy, since Nup192-yEGFP foci are also less targeted to the vacuole of *atg15*Δ cells in the absence of *BFA1* (Supplementary Figures S5A-B).

Reviewer #2:

The authors have made important improvements to the manuscript and have done a good job at solidifying data supporting an interaction between Bfa1 and Nup159. There remains considerable doubt, however, as to whether this interaction plays any role in nuclear pore clearance by autophagy. New evidence presented, for example the genetic interaction between *NUP159* and *BFA1* in the presence of rapamycin, are interesting but without a direct examination of nup degradation, this experiment is open to interpretation. Likewise, although GFP-Atg8 may be less efficiently degraded in *nup159-1* cells, the relationship to NPC/nup turnover remains ill defined. As it stands, the definitive Nup159/133-GFP fallout experiments do not demonstrate any clear difference in the degradation of nups in the absence of BFA1 during nitrogen starvation. The authors suggest that this may be due to the presence of Nup159-independent routes to deliver NPCs to the vacuole such as Atg39-dependent nucleophagy and piecemeal microautophagy of the nucleus but neither of these pathways have been demonstrated to degrade whole NPCs and it would have been straightforward to introduce mutants to perturb these pathways. Thus, the strong suggestion is to amend the title to be more circumspect with respect to the role of a Bfa1-Nup159 interaction in NPC phagy.

We thank the reviewer for acknowledging our effort to solidify the data in our manuscript, to which it greatly contributed the suggestions made by both referees during the original revision of our work. We believe that our results firmly support a role of Bfa1 in the Nup159-mediated autophagy pathway, initially described by the Nakatogawa and Pfander labs (Tomioka *et al.*, 2020; Lee *et al.*, 2020). In this sense, we now also show that expression of the *nup159-1* allele induces *ATG8* expression and, hence, the activation of autophagy (Figure for Reviewers A). Together with a less efficient degradation of GFP-Atg8 in *nup159-1* cells lacking Bfa1, this further supports that the phenotypes showed by *bfa1*Δ cells are indeed linked to a reduced function of the Nup159-dependent autophagy pathway. In any case, we also appreciate the point that the reviewers raise about as to whether Bfa1 and Nup159 specifically collaborate to promote NPC clearance or they rather cooperate in other undetermined Nup159-dependent autophagic processes. The purpose of the experiments with the *atg39*Δ mutant was exclusively to demonstrate that the defect shown by the lack of Bfa1 is similar to that displayed by mutants in other autophagic pathways in the presence of aggregates of damaged NPCs. We chose Atg39 because it was proposed to be a major pathway for nucleoporin degradation together with Nup159 (Tomioka *et al.*, 2020). However, the fact the we do not see a synergistic defect of the *ATG39* and *BFA1* gene deletions after autophagy is induced with rapamycin and cells are further challenged with an accumulation of misfolded nucleoporin aggregates likely suggests that, in fact, Atg39-dependent nucleophagy is not the main pathway that substitutes for the lack of Bfa1. Indeed, in agreement with our previous observations, we have followed the reviewer's suggestion and found no synergistic defects in the degradation of GFP-Atg8 in *bfa1*Δ cells after the Atg39-dependent pathway was further impaired when autophagy was specifically induced by NPC damage caused by expression of the *nup159-1* allele (Figure for Reviewers B-C).

Multiple pathways have been involved by different laboratories in the clearance of nucleoporins and/or NPCs from the nuclear envelope, including not only Nup159-mediated autophagy, Atg39-dependent nucleophagy or piecemeal microautophagy, but also the ubiquitin-proteasome system, an ESCRT-III/Vps4-dependent mechanism that plays a role in potentially recognizing and clearing defective NPC assembly intermediates or a compartment termed the SINC that sequesters defective NPCs to prevent their passage to daughter cells (Webster *et al*., 2014; Lee *et al*., 2020; Tomioka *et al.*, 2020; Allegretti *et al.*, 2020; Yin and Klionsky, 2020). We have modified the text to incorporate the reviewer's comments regarding the pathways that control the clearance of nuclear pores by autophagy. However, a fundamental issue that complicates the analysis of this process is that we are still far to understand how cells specifically deal with non-functional NPCs or damaged nucleoporin subcomplexes that aggregate in the nuclear envelope (Webster *et al.*, 2014). The relative contribution and the specific target (whole NPCs, nucleoporin complexes or individual nucleoporins) of each of the different pathways that seem to participate in NPC quality control have not been sufficiently clarified. Moreover, one can find abundant contradictory data in the bibliography regarding their precise role. In this way, and exclusively focusing on Nup159, while Tomioka *et al*. proposed that Atg39-dependent nucleophagy and the Nup159-dependent autophagy are the two main pathways for nucleoporinophagy (Tomioka *et al.*, 2020), Lee *et al*. discarded a role for Atg39 and instead proposed a model for NPC quality control by which the number and/or integrity of NPCs is controlled by the proteasome and the Nup159-dependent autophagic machinery (Lee *et al.*, 2020). Furthermore, it has been more recently demonstrated that Nup159 participates in a previously undescribed autophagic pathway that is independent of known nucleophagy mechanisms, receptor proteins and the Atg11 scaffold, and that targets certain transcriptional regulators for rapid vacuolar proteolysis, utilizing the RNA remodeling complex, the sorting nexin heterodimer Snx4-Atg20, Atg17, and the core autophagic machinery (Hanley *et al.*, 2021). Hence, Nup159 seems to play a role, not only in the degradation of nucleoporins (Tomioka *et al*., 2020) or nuclear pore complexes (Lee *et al.*, 2020), but also in other autophagic processes unrelated with the clearance of damaged nuclear pore components.

Taking all of above into consideration, and to hopefully satisfy the reviewers' concerns regarding the functional significance of the Nup159-Bfa1 association, we have finally opted to exclusively conclude that Bfa1 participates in the Nup159-dependent autophagy pathway, a statement that we believe that our experiments firmly support. Accordingly, we have toned down our claims concerning the specific function of Bfa1 and Nup159 in nuclear pore clearance, which we only maintain as an interesting possibility to be further explored and substantiated in the future. Finally, and as additionally requested by the reviewer, we have also changed the title of our manuscript to be more circumspect to the new message that we want to convey.

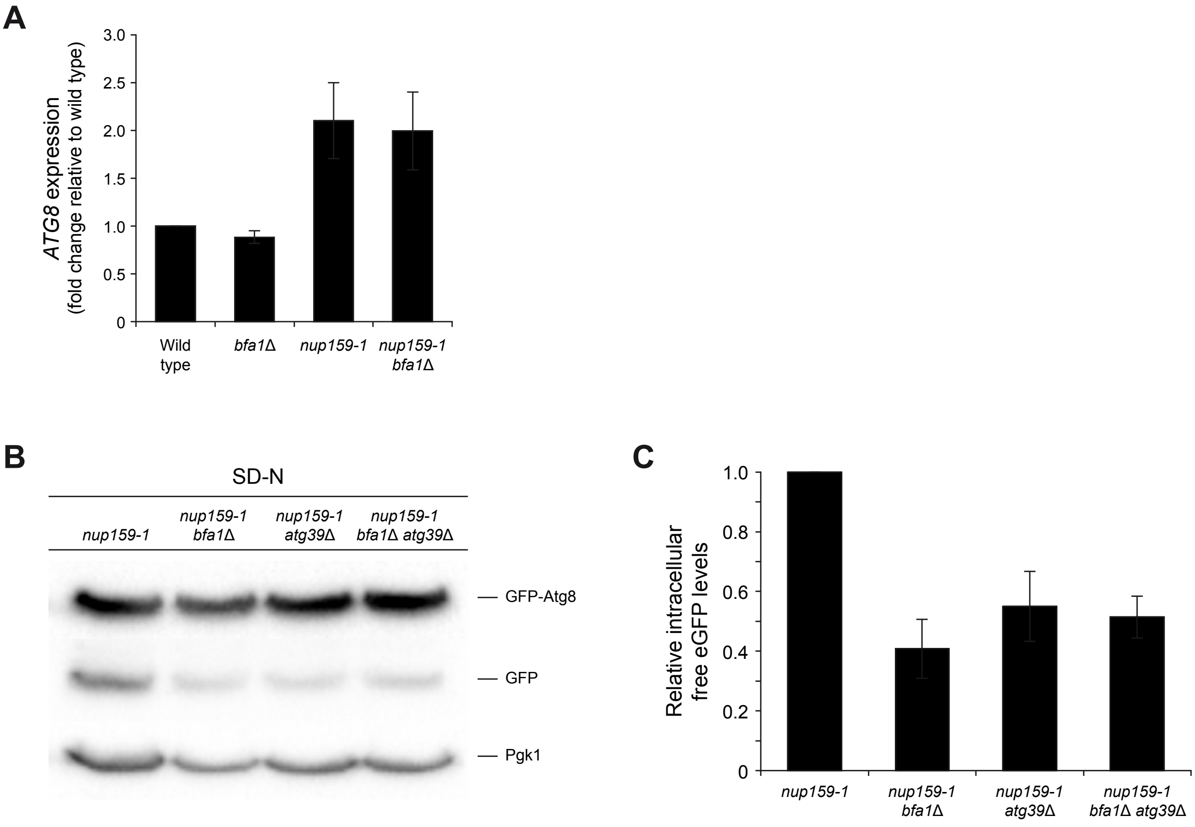

Figure for Reviewers
